# Temperature and feeding induce tissue level changes in autotrophic and heterotrophic nutrient allocation in the coral symbiosis – A NanoSIMS study

**DOI:** 10.1038/s41598-018-31094-1

**Published:** 2018-08-23

**Authors:** Thomas Krueger, Julia Bodin, Noa Horwitz, Céline Loussert-Fonta, Adrian Sakr, Stéphane Escrig, Maoz Fine, Anders Meibom

**Affiliations:** 1 Laboratory for Biological Geochemistry, School of Architecture, Civil and Environmental Engineering, Ecole Polytechnique Fédérale de Lausanne (EPFL), CH-1015 Lausanne, Switzerland; 2 The Mina and Everard Goodman Faculty of Life Sciences, Bar-Ilan University, Ramat-Gan, 52900 Israel; 3 The Interuniversity Institute for Marine Sciences, Eilat, 88103 Israel; 4 Electron Microscopy Facility, University of Lausanne, Biophore, CH-1015 Lausanne, Switzerland; 5 Center for Advanced Surface Analysis, Institute of Earth Sciences, University of Lausanne, CH-1015 Lausanne, Switzerland

## Abstract

Corals access inorganic seawater nutrients through their autotrophic endosymbiotic dinoflagellates, but also capture planktonic prey through heterotrophic feeding. Correlating NanoSIMS and TEM imaging, we visualized and quantified the subcellular fate of autotrophic and heterotrophic C and N in the coral *Stylophora pistillata* using stable isotopes. Six scenarios were compared after 6 h: autotrophic pulse (^13^C-bicarbonate, ^15^N-nitrate) in either unfed or regularly fed corals, and heterotrophic pulse (^13^C-, ^15^N-labelled brine shrimps) in regularly fed corals; each at ambient and elevated temperature. Host assimilation of photosynthates was similar under fed and unfed conditions, but symbionts assimilated 10% more C in fed corals. Photoautotrophic C was primarily channelled into host lipid bodies, whereas heterotrophic C and N were generally co-allocated to the tissue. Food-derived label was detected in some subcellular structures associated with the remobilisation of host lipid stores. While heterotrophic input generally exceeded autotrophic input, it was more negatively affected by elevated temperature. The reduced input from both modes of nutrition at elevated temperature was accompanied by a shift in the partitioning of C and N, benefiting epidermis and symbionts. This study provides a unique view into the nutrient partitioning in corals and highlights the tight connection of nutrient fluxes in symbiotic partners.

## Introduction

The immense biodiversity and productivity of tropical coral reefs exists in a marine environment that is generally characterized by low nutrient levels and plankton concentrations. Coined “Darwin’s paradox”, the investigation into why corals thrive in areas with mostly low nutrients has directed the scientific focus towards nutrient fluxes and cycles within the overall reef framework and within individual hermatypic corals^1,2^. While corals obtain dissolved organic nutrients from the seawater^3,4^, the coral animal is also given unique access to the inorganic nutrient pool through endosymbiotic photosynthesizing microalgae (mainly *Symbiodinium* sp.) and via parts of its microbial community, such as cyanobacteria and diazotrophic bacteria^5,6^. Intracellular *Symbiodinium* sp. cells photosynthesize in the light and fix CO_2_ into triose phosphate compounds that can serve as carbon skeletons for subsequent processes, such as inorganic N assimilation and the formation of amino acids^7^. The CO_2_-fixation rate in *Symbiodinium* is very high and C is rapidly assimilated into sugars and amino acids within seconds to minutes^8,9^. The translocation of part of these soluble photosynthates from the symbiont to the animal tissue happens across the entire colony. While it has been demonstrated that symbiont autotrophy can be sufficient to meet the metabolic C demands of the coral animal^10,11^, coral heterotrophy (e.g. hunting for planktonic prey) is an important component of coral nutrition, especially in cryptic and mesophotic habitats or turbid water environments, where light becomes a limiting factor^12–14^. Upon loss of autotrophic input, as is the case in bleached corals, heterotrophy becomes fundamental for survival and recovery of the coral^15,16^. Under normal, healthy conditions, the relative contribution of autotrophy and heterotrophy to coral nutrition is modulated by the environment and may vary between species^17^. Importantly, the intracellular microalgae also allow for a reassimilation of metabolic ‘waste’ products of the animal host (e.g. NH_4_^+^ and CO_2_)^18^. Hence, the symbiotic nature of the coral animal ensures access to organic and inorganic nutrients as well as recycling and conservation of acquired nutrients in an oligotrophic environment.

Despite their autotrophic capability that originated in the middle to late Triassic^19–21^, symbiotic scleractinian corals have retained highly developed feeding mechanisms (behavioural and anatomical features) that characterize them as heterotrophic organisms. Heterotrophic nutrient sources include dissolved and particular organic matter as well as a range of plankton sizes ranging from pico- to meso- and macrozooplankton^22^. Coral polyps actively prey on zooplankton using cnidocyte-containing tentacles and/or mesenterial filaments^23^. Indeed, coral recruits have been observed to capture zooplankton as early as 8 days after settlement^24^. Ciliary currents directed towards the mouth or the generation and re-ingestion of mucus nets and strings on the surface assist in prey capture^25,26^. All coral polyps in colonial corals are connected by coenenchyme tissue (*sensu* coenosarc tissue) and their gastrovascular cavities share a network of gastrovascular canals that serves as a circulatory system for transporting material^27–29^. Despite their wide-ranging gastrovascular system, corals lack specialized tissues for storing molecules that fuel metabolism. Instead, the coral tissue contains a high density of transitory C storage bodies, such as lipid bodies and small granular glycogen deposits that all receive photoautotrophic C^9,30,31^.

Nutrient assimilation serves two purposes: to generate energy (catabolism) or to build cellular and tissue structures (anabolism). Excretion of metabolic waste products and secretion of mucus as well as reproductive output constitute the main pathways for the loss of nutrients in corals^30,32^. Quantifying and modelling the partitioning of nutrients from autotrophic and heterotrophic pathways between host and symbiont has traditionally been achieved through measurements of radioactive or stable isotopes in bulk tissue samples^5,33–37^. Recently, nanoscale secondary ion mass spectrometry (NanoSIMS) analysis has provided ultrastructural information on the fate of nutrients when correlated with transmission electron microscopy (TEM) by mapping the subcellular distribution of elements and their stable isotopes. This technique has added a visual dimension to autotrophic coral nutrition and has captured the dynamic nature of nutrient fluxes within and between different coral tissue compartments^9,38–42^.

Here, we identified the subcellular sinks of autotrophic and heterotrophic nutrients in the surface body wall of the coral coenenchyme in the cosmopolitan, symbiotic coral *Stylophora pistillata* (Red Sea specimen). We specifically investigated to what extent heterotrophic nutrients are shared between both partners and whether regular feeding of the host affects the autotrophy of its symbiont population with regard to inorganic C and N assimilation and translocation to the host. Compartment-specific assimilation rates and partitioning of C and N between symbiont, gastrodermis, and epidermis were contrasted between autotrophic and heterotrophic mode of nutrition. Furthermore, we assessed how prolonged thermal exposure in this otherwise temperature-resistant coral^42,43^ modulated the nutritional symbiotic relationship.

## Results and Discussion

### The ultrastructural fate of autotrophic C and N

**Figure 1:**
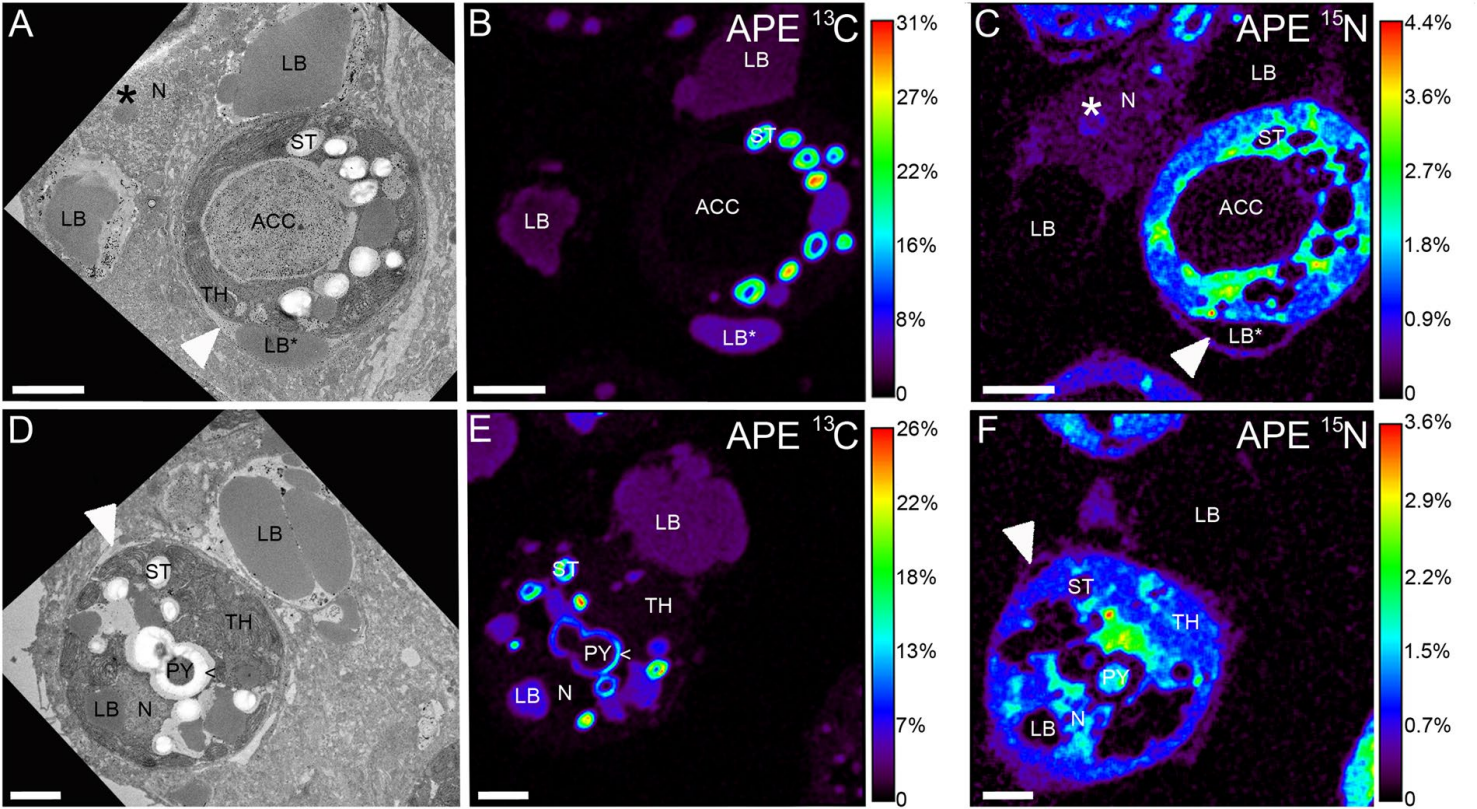
Autotrophic uptake of carbon and nitrogen. Correlated TEM and NanoSIMS images of the distribution of ^13^C and ^15^N in *Symbiodinium* and surrounding gastrodermal cell cytoplasm, resulting from simultaneous photosynthetically driven assimilation of [^13^C]bicarbonate **(B**,**E)** and [^15^N]nitrate **(C**,**F)** after a 6 h isotopic pulse in the light. Fixed ^13^C and ^15^N were detected throughout the symbiont cell, with carbon mainly concentrated in the primary starch sheet (<) around the dinoflagellate pyrenoid (PY) near thylakoid membranes (TH), in secondary starch granules (ST), symbiont and host lipid bodies (LB), and in extra-algal lipid bodies (LB*). Symbiont nitrogen labelling was lowest in carbon-rich structures and the accumulation body (ACC), and highest in small crystalline-like hotspots. Translocated ^13^C to the host tissue was mainly concentrated in lipid bodies, whereas nuclei (N) and nucleoli (*) in the host cell showed high ^15^N labelling. Colours in NanoSIMS maps represent enrichment relative to an unlabelled sample, expressed as atom percent excess (APE). White arrows **(A**,**C**,**D**,**F)** point to symbiosome membrane. Scale bars are 2 µm.

Consistent with previous studies in other corals^38,40^, fixed and assimilated DIC was primarily deposited in primary and secondary starch deposits and lipid bodies in the symbiont, and as translocated product in the host lipid bodies of the coral gastrodermis (Fig. 1A,B,D,E). Occasionally detected extra-algal lipid droplets, located outside of the algae but within the symbiosome, showed equally high levels of C turnover (Fig. 1A,B: LB*), confirming the release of photosynthetically derived lipids into the host vacuole^9,44,45^. Fixed nitrate was incorporated relatively homogenously throughout the symbiont cell (Fig. 1C,F). Occasional intracellular ^15^N hotspots were similar to previously characterized uric acid crystals in symbiotic cnidarians^40,46^ and likely serve as highly concentrated transitory N stores within *Symbiodinium*. The lowest levels of photosynthetic C and N incorporation in the symbiont were observed for the accumulation body (Fig. 1B,C).

**Figure 2:**
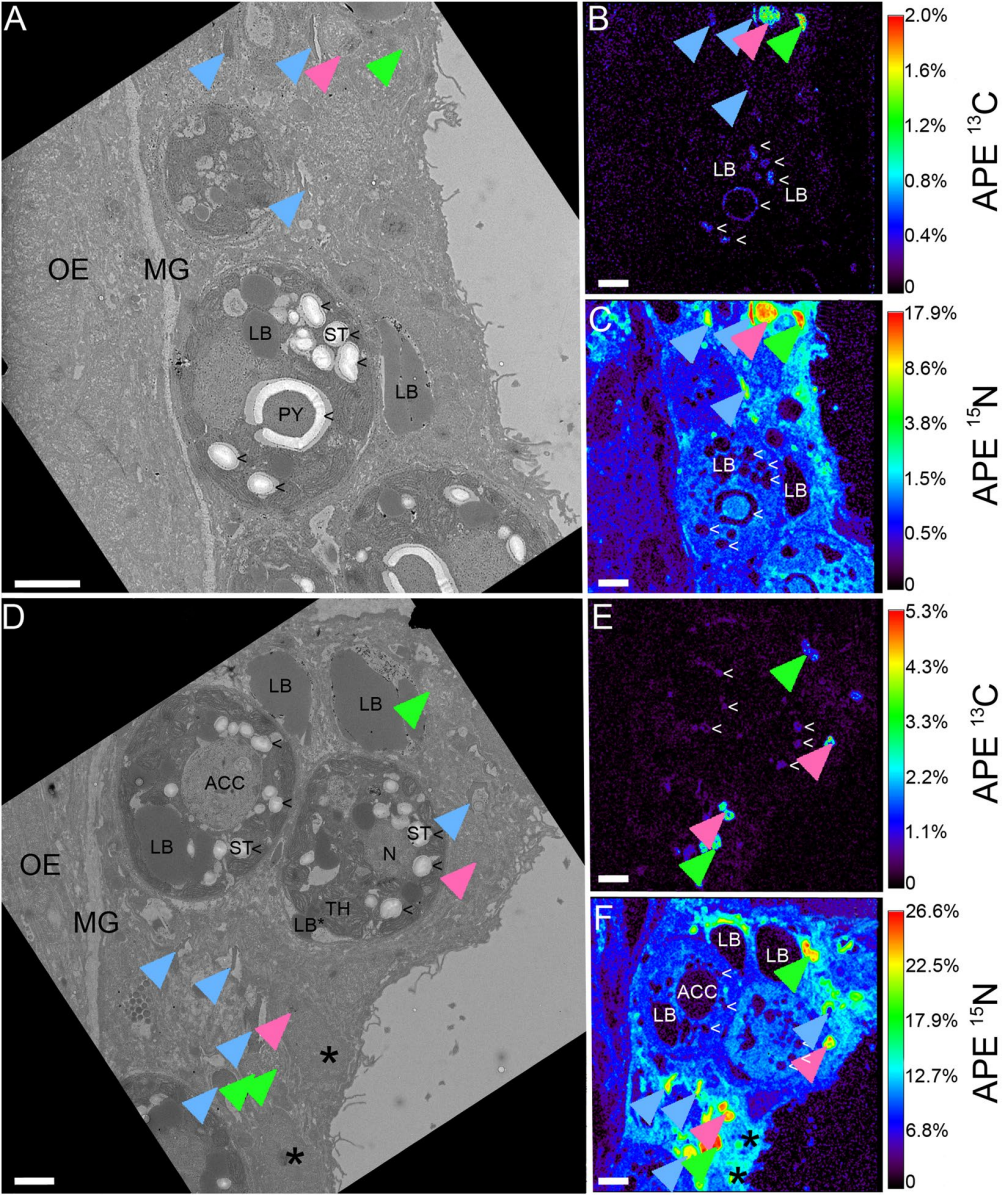
Heterotrophic carbon and nitrogen assimilation of coral gastrodermis. Correlated TEM and NanoSIMS images of the distribution of prey ^13^C **(A**,**E)** and ^15^N **(C**,**F)** in the surface body wall gastrodermis 6 h after ingestion of labelled *Artemia salina* began. Subcellular features abbreviated as in Fig. 1, with OE indicating the surface body wall epidermis and MG the mesoglea. Multiple gastrodermal ^13^C and ^15^N hotspots were observed (coloured arrows, detailed in Figs. 3, 4). Hotspots of ^13^C in the symbionts corresponded to starch (<). Colours in NanoSIMS maps represent enrichment relative to an unlabelled tissue as atomic percent excess (APE) (note log scale in **C,F**). Asterisks indicate host nucleoli. Scale bars are 2 µm.

The host tissue showed variable levels of labelling with autotrophic C and N. While gastrodermal lipid bodies served as the main C stores for the coral host (Fig. 1A,B,D,E), host gastrodermal labelling with autotrophic N was uniform and likely reflected the general protein turnover in the host tissue. Differential labelling of subcellular organelles with ^15^N was only distinguishable in the immediate vicinity of the symbionts, where host nuclei and nucleoli showed significant incorporation (Fig. 1C). Especially host nucleoli showed a high turnover for autotrophic and heterotrophic N (asterisks in Figs 1A,C and 2D,F), likely as result of the faster turnover of nucleolar ribosomal proteins relative to the nuclear structure^47^. Interestingly, the symbiosome membrane that encapsulates the symbionts in their host-derived vacuole incorporated a significant amount of symbiont-derived N within the pulse period (Fig. 1C,F arrow). This rapid turnover of N-containing components (e.g. membrane proteins) reveals a highly dynamic nature of the symbiosome membrane that is fuelled by the photosynthetic activity of its occupant.

### The ultrastructural fate of heterotrophic C and N

**Figure 3:**
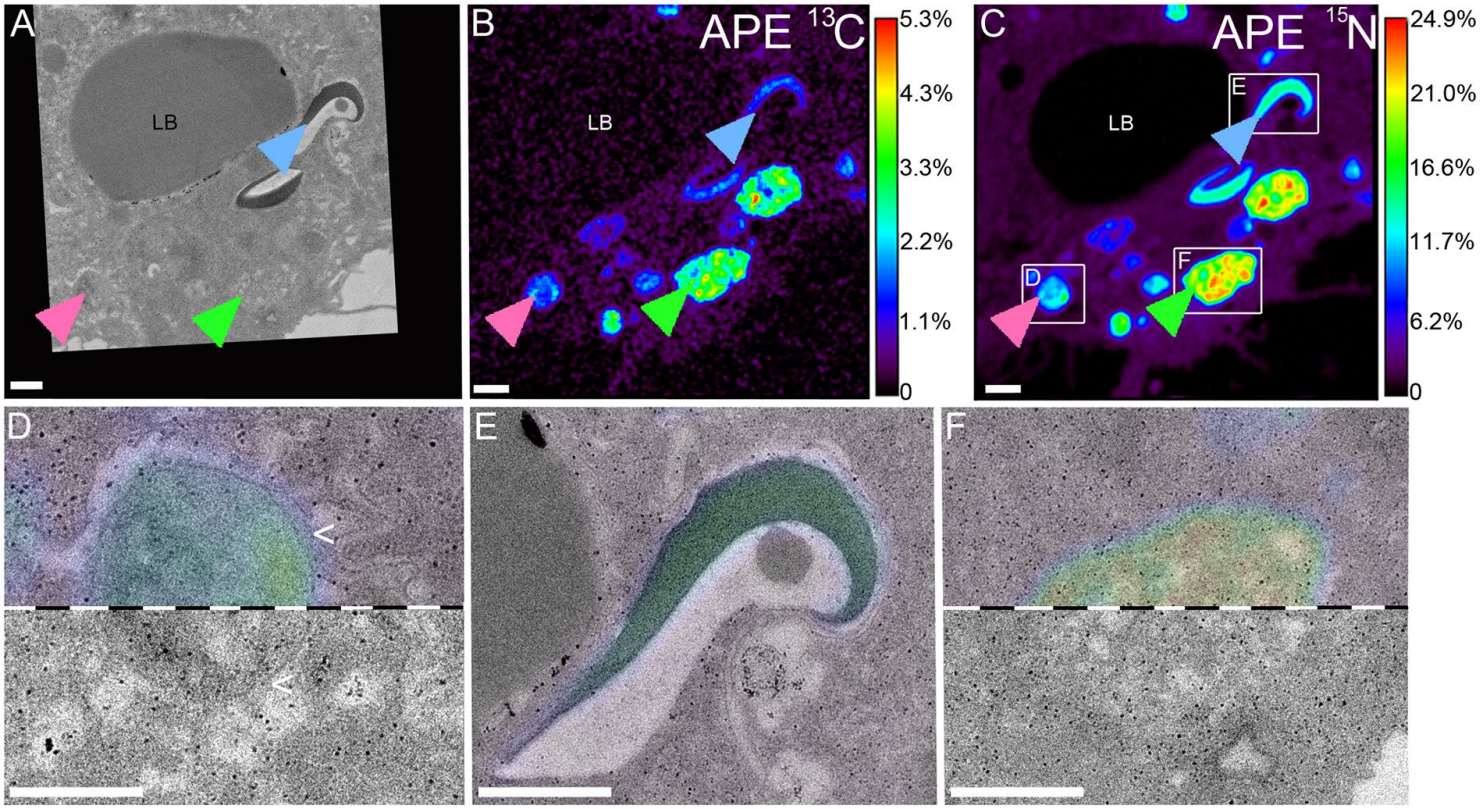
Types of isotopic hotspots in the gastrodermis from heterotrophy. Correlated TEM and NanoSIMS images for ^13^C **(A**,**B)** and ^15^N **(A**,**C)** highlighting three types of isotopic hotspots (coloured arrows; see text) with TEM ultrastructure and partial ^15^N-signal overlay **(D–F)**. Note the presence of a membrane around the hotspot in D (>). Colours in **(B**,**C)** display enrichment relative to an unlabelled tissue as atom percent excess (APE). Scale bars are 1 µm **(A–C**, **E**,**F)** and 750 nm **(D)**.

**Figure 4:**
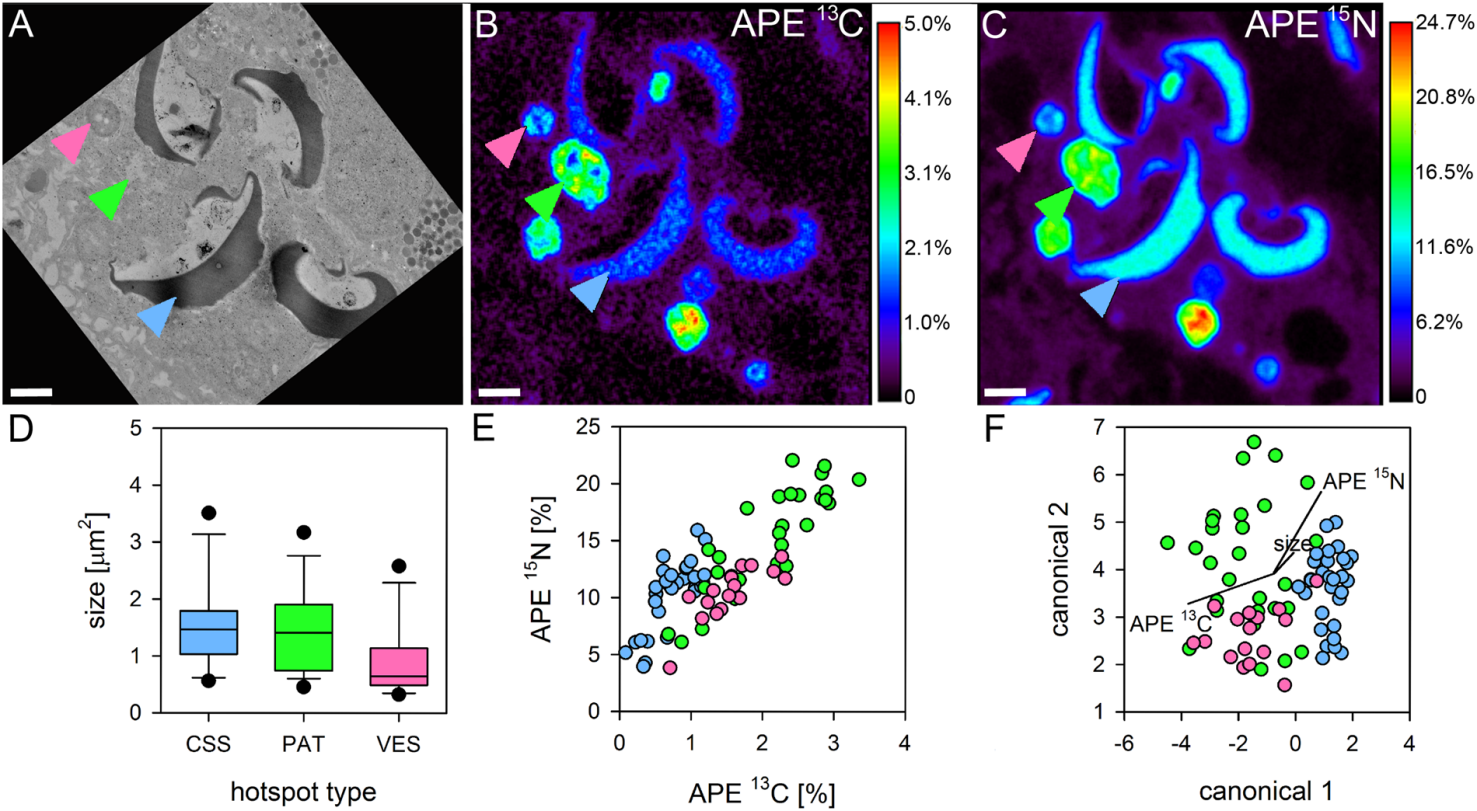
Characteristics of isotopic hotspots in the gastrodermis from heterotrophy. Multiple high-resolution (15 × 15 µm) TEM and NanoSIMS images **(A–C)** were analysed for size **(D)** and individual enrichment **(E)** of the three types of isotopic hotspots in the gastrodermis. CSS: cup-shaped structures (blue arrows), PAT: electron-lucent patches (green), VES: vesicles (pink). Canonical correlation analysis of these characteristics revealed a distinct CSS cluster with overlapping PAT and VES clusters **(F)**. Colours in **(B)** and **(C)** display enrichment relative to an unlabelled tissue as atom percent excess (APE). Scale bars are 1 µm.

**Figure 5:**
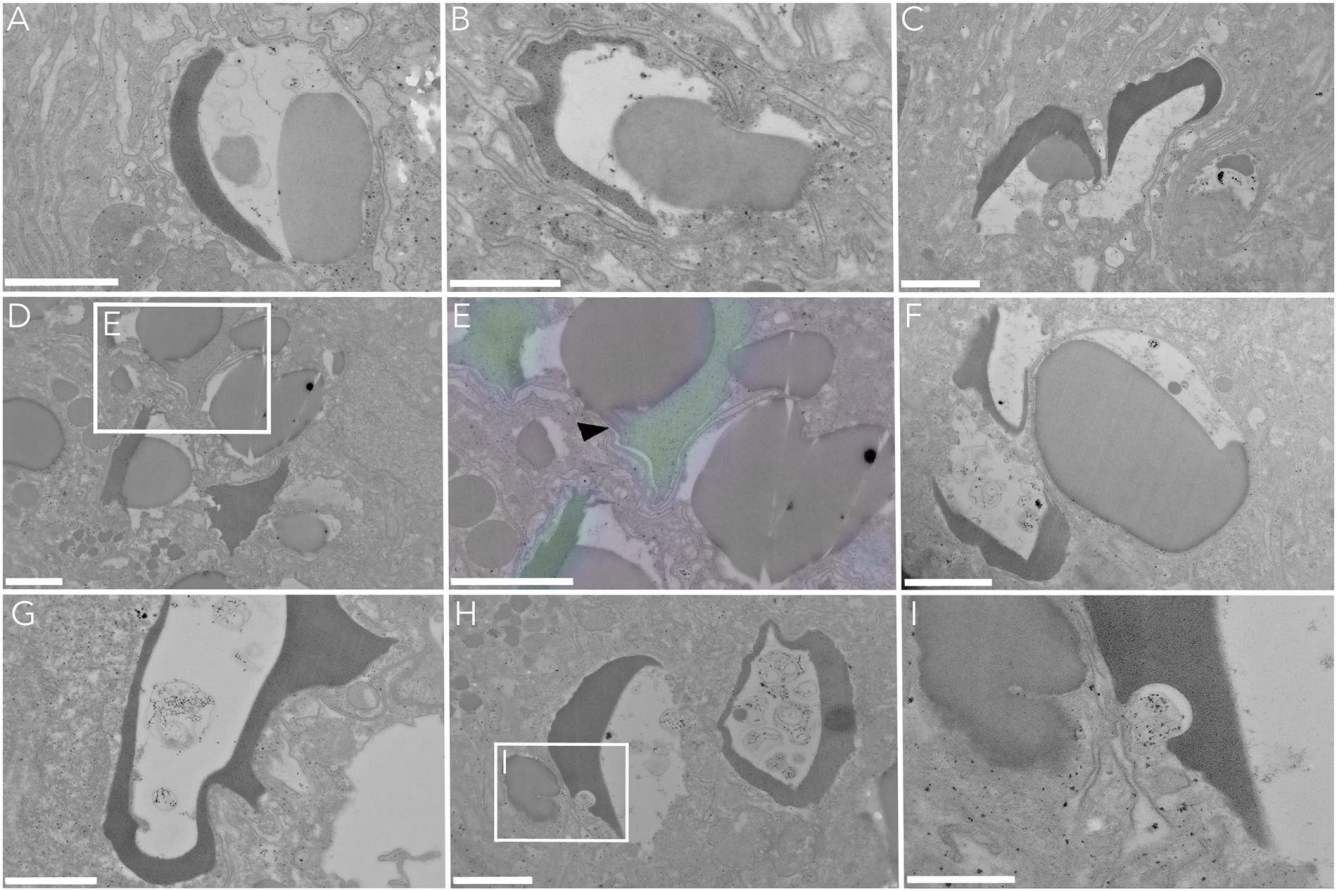
Association of CSS with gastrodermal host lipid bodies. Cup-shaped electron-dense structures that were highly enriched with heterotrophic C and N (cf. Fig. 4) were consistently found next to partially extracted host lipid bodies **(A**,**B)** or in direct contact with them **(C–E)**. E shows the formation of a membrane continuity between both structures, despite the distinct separation of the isotopic signal (black arrow in E with ^15^N-signal as colouration). Areas with extracted lipids consistently showed the presence of residual material **(F–H)**. Intrusion of the host ER membrane into CSS **(I)**. Scale bars are 1 µm **(A**,**C–H)** or 500 nm **(B**,**I)**.

Heterotrophic ^13^C and ^15^N derived from the digested brine shrimp was detected in all coral tissue layers after 6 h. Notably, we detected substantial enrichment from heterotrophic food also in the basal body wall gastrodermis and the adjacent calicodermis (Fig. S1A–C), illustrating a direct contribution of heterotrophic nutrients to the cellular activity of the tissue layer responsible for calcification. Enrichment was highest in the gastrodermis for both elements (Fig. 2), where numerous gastrodermal hotspots (~1–2 µm diameter) expressed strong co-labelling of C and N. Three distinct types of labelled hotspots could be differentiated in the gastrodermis: (i) round, membrane-delineated vesicles (VES) that contained homogenous labelled material (pink arrows, Figs 2, 3D, 4 and S1A–C), (ii) elongated, electron-dense cup-shaped structures (CSS) that were in close proximity with the host lipid bodies (blue arrows, Figs 2, 3E, 4, 5 and S1A–C), and (iii) oval or elongated electron-lucent patches (PAT), which were ultrastructurally similar to the host cytoplasm (green arrows, Figs 2, 3F, 4 and S1A–C). With regard to their isotopic signature, VES and PAT formed a continuum, whereas CSS tended to be more enriched in ^15^N relative to ^13^C (Fig. 4D–F). These three types of gastrodermal hotspots likely reflect different stages of food processing with VES acting as transitory structures that contain endo- or phagocytosed food material. Cup-shaped structures appear to be more permanent ultrastructural features and could be regularly found in *S*. *pistillata*, but have also been described in larvae and adult tissue of *P*. *damicornis*^48,49^. Their close/direct contact with host lipid bodies (Fig. 5A–E) and their rapid incorporation of heterotrophic N (this study) and external ammonia^48,49^ suggest a crucial function in coral lipid metabolism, potentially related to the remobilization and/or breakdown of lipid droplets as evidenced by the presence of residual material in the vicinity of the extraction (Fig. 5F–H). In one case, we even observed a membrane continuity between CSS and lipid bodies, despite a clear spatial separation of the isotopic signal (Fig. 5D,E). The specific function of PAT ^15^N-hotspots remains unclear. Either, the high proportion of heterotrophic ^13^C and ^15^N identifies them as unprocessed food remnants or these patches are indeed areas where new host cellular material is rapidly formed, incorporating the labelled elements. Since no clear TEM ultrastructure and no dedicated cellular machinery (e.g. ER, mitochondria) was found in the immediate surrounding (Fig. 3F) and such highly enriched gastrodermal hotspots were not found in the autotrophic treatment, we discount the possibility of dedicated cytoplasmic areas with permanently high C and N turnover. Due to their similar isotopic signatures (Fig. 4E,F), patches potentially represent released material from vesicles, but further work is required.

Few isotopic hotspots were also found in the host epidermis, representing mostly small vesicles, but with a much lower level of enrichment than the gastrodermal vesicles (Fig. S1D–F). Heterotrophic N-labelling in the symbiont cell was homogenous, although occasional small vesicle-like hotspots could be observed (Figs 2C,F and S2) similar to the autotrophic mode of nutrition. Detectable heterotrophic C-labelling was restricted to starch granules and the primary starch sheet around the pyrenoid (Fig. 2B,E).

### Modulation of symbiont autotrophy by regular holobiont feeding and elevated temperature

**Figure 6:**
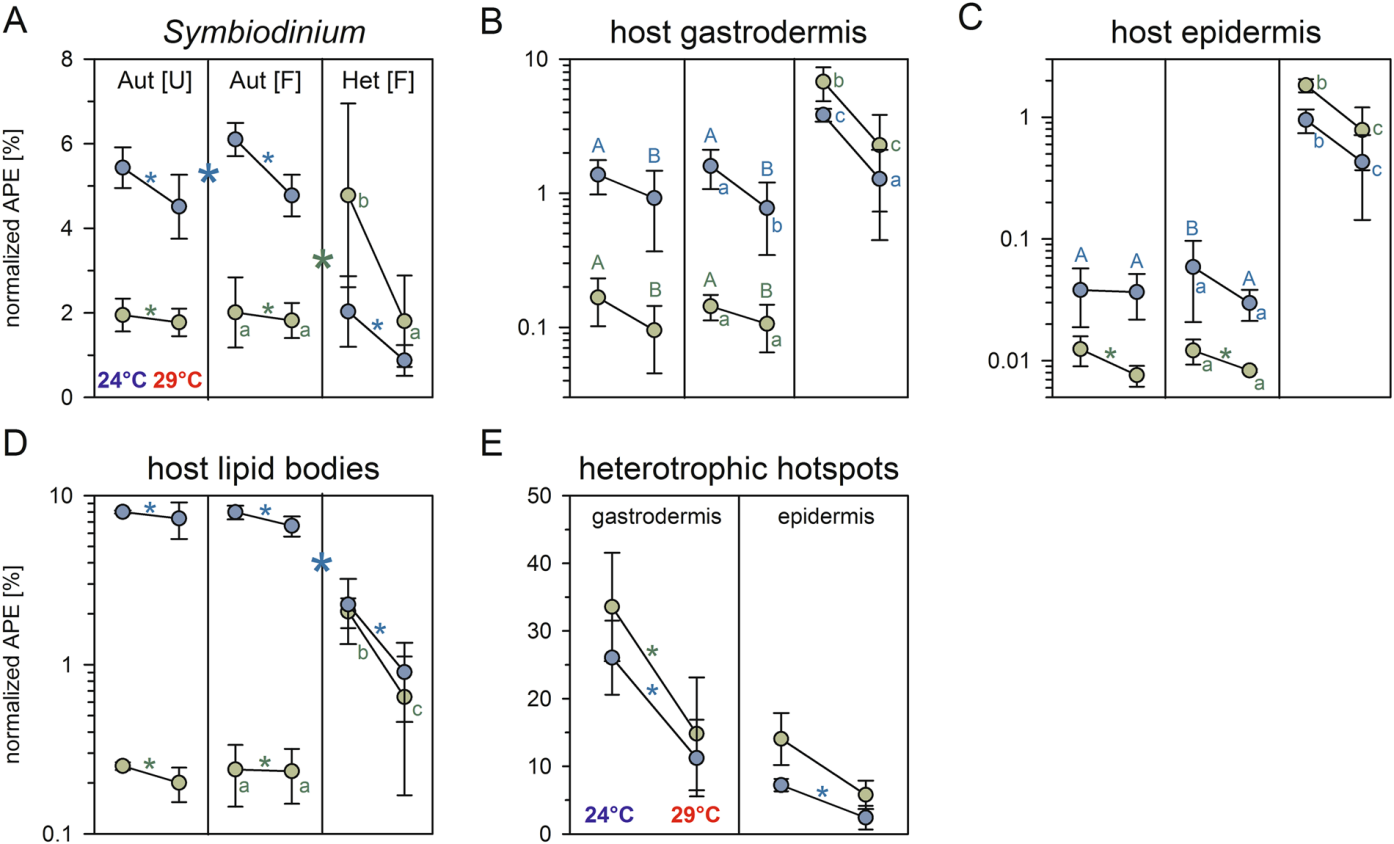
Compartment-specific carbon and nitrogen turnover. Carbon (blue) and nitrogen (green) turnover in different compartments of the surface body wall of the coral coenenchyme in Northern Red Sea *Stylophora pistillata* at the end of a 6-week exposure period to ambient (24 °C; left within columns) or elevated SST (29 °C; right; cumulative exposure was 11.2 DHW). Mode of nutrition indicated as autotrophic assimilation (Aut) of bicarbonate and nitrate or heterotrophic assimilation (Het) of *Artemia salina* prey in unfed [U] or regularly fed corals [F; twice a week for 67 days]. Depicted enrichment data are normalized to the labelling level in each food source to allow comparison (see Methods) and thus represents the proportion of the total C or N pool that has been replaced with newly assimilated ^13^C and ^15^N, assuming steady state conditions. Aut [U] dataset represents amended dataset from Krueger *et al*.^42^. Note log scales in B, C, D. For simplicity, data points depicted here are means of three tested colonies (±SD; Table S4), while statistical results reflect the nested nature (multiple data points per colony and treatment; individual N_*Symbiodinium*_ = 40–60, N_gastrodermis/epidermis_ = 10–15, N_lipid bodies_ = 40–70, N_hotspots_ = 30–250) and paired nature (same colonies in all treatments) of the data (detailed colony-specific responses in Fig. S4, Table S4). (**A–D**): Either significant main effects of temperature (* within columns), feeding acclimation (* between treatments), and/or interactive effects (letters based on Tukey HSD *post hoc* results) are indicated for Aut [U] *vs*. Aut [F] (capital letters; Table S2) and Aut [F] *vs*. Het [F] (small letters; Table S3) comparisons. Treatments not connected by the same letter are significantly different. (**E**) Statistical results for heterotrophic hotspot enrichment are based on one-sided paired t-tests (N = 3), where all data points within each replicate were reduced to a mean value due to the strong imbalance in N between thermal treatments (Table S4). Gastrodermal hotspots: ^13^C: t(2) = −9.173, p = 0.0058*; ^15^N: t(2) = −6.297, p = 0.0122*; Epidermal hotspots: ^13^C: t(2) = −4.213, p = 0.0232*; ^15^N: t(2) = −2.689, p = 0.0575.

After 6 h of photosynthetic activity under ambient conditions, the symbionts replaced on average 5–6% of their structural C (from dissolved inorganic carbon [DIC]) and ~2% of their N (from nitrate) (Fig. 6A). Regular feeding of corals supplements the symbiont with limiting elements such as N and P^22,50,51^, but also increases host tissue thickness and symbiont densities that alter the light microenvironment for individual cells^22^. While such tendencies were also observed here (Table S1), symbionts in regularly fed corals showed significantly increased assimilation of autotrophic C (+10 ± 8%), but not N, for both temperatures (mean ± SD, N = 6, Fig. 6A, Table S2). Such a positive effect was also seen for host epidermal C turnover, albeit much more variable between colonies and only at ambient temperatures (+51 ± 57%; N = 3; Fig. 6C, Table S2).

Assuming that the relationship between host anabolism and catabolism was not fundamentally different between unfed and fed corals, this extra amount of photosynthetically assimilated C was primarily utilized by the symbionts themselves, matching previous bulk observations for this coral^14^. Thus, the uncoupling of photosynthesis and growth under nutrient limitation is eased under regular host feeding, confirming the expected effect that the symbionts retain a larger share of their fixed C for their individual cell and population growth^52^.

The absence of a similar impact on N assimilation might be attributed to the isometric increase in areal host and symbiont biomass, leaving symbiont density per host protein unchanged (Table S1). Thus, the tendency for increased areal ammonia production due to more tissue that might have shifted the N prevalence of the *in hospite* population from nitrate to the preferred ammonia^53–58^ (as is prevalent in anemones^59^) was likely offset by higher symbiont numbers (p = 0.053, Table S1). With the exception of carbon in the epidermis, feeding state of the holobiont did not significantly modify the negative impact of elevated temperature on the autotrophic acquisition of C or N (Fig. 6, Table S2).

### Autotrophic vs. heterotrophic nutrient assimilation in the coral host

**Figure 7:**
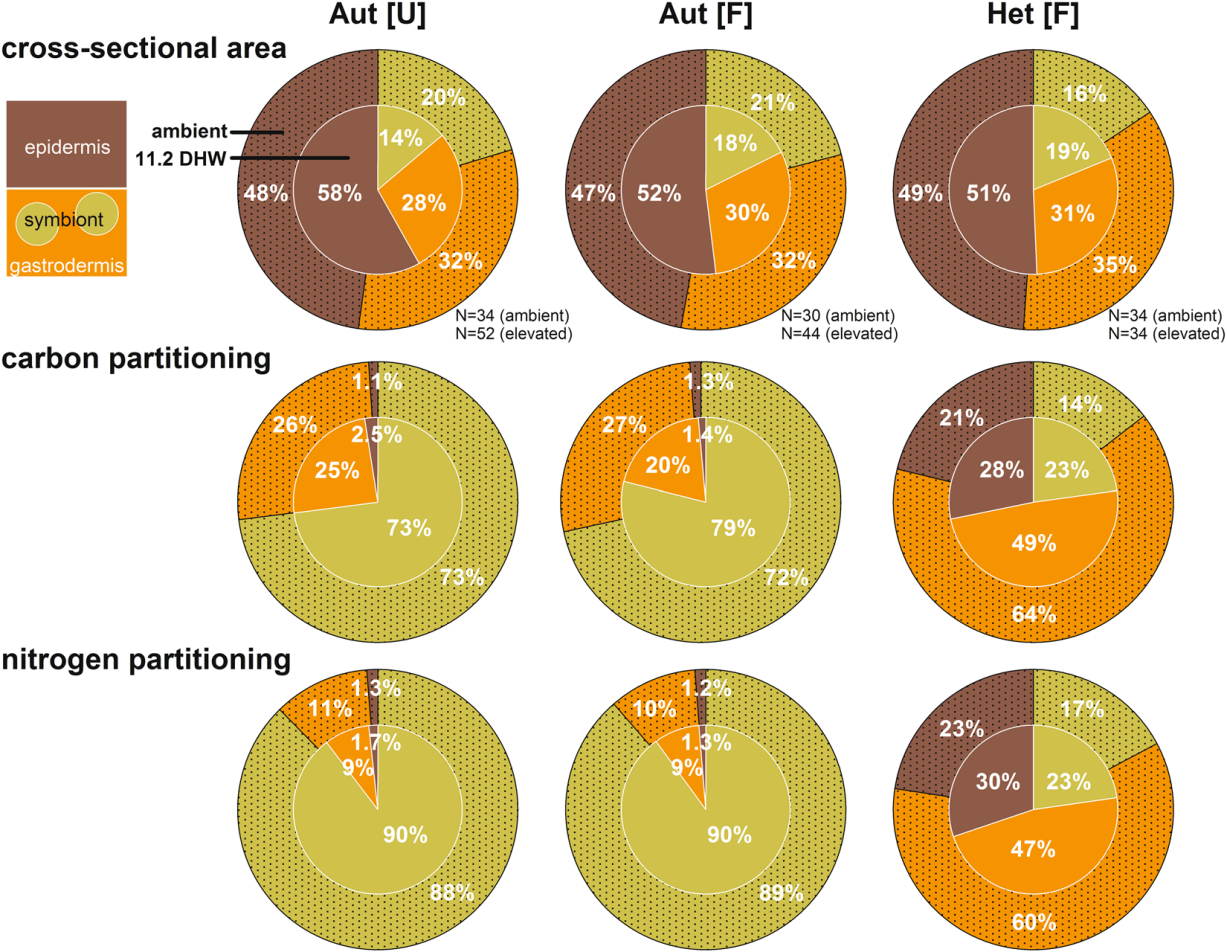
Partitioning of assimilated ^13^C and ^15^N in the coral symbiosis after 6 hours. Shown are the shares of the total cross-sectional area composed of symbiont (yellow), host gastrodermis (orange), and host epidermis (red) of the surface body wall coenenchyme and the ^13^C- and ^15^N distribution across these compartments in each mode of nutrition (pie charts left to right) at ambient conditions (outer filled part) and after 11.2 DHW (inner clear part). Data for C and N partitioning were obtained by combining the size of each compartment with its average ^13^C and ^15^N turnover (Fig. 6) obtained from multiple NanoSIMS images (N indicated; see also IAF calculation in Methods). These data provide a semi-quantitative view on the distribution of the assimilated C- and N-pool at the end of the 6 h pulse.

Host assimilation of C and N was strikingly different in terms of turnover (Fig. 6) and allocation (Fig. 7) between both modes of nutrition. Average autotrophic nutrient assimilation in the gastrodermis displayed a strong bias towards C turnover (1.0–1.6%) compared with a low N turnover (0.1–0.2%) after 6 h (Fig. 6B,D). In contrast, the influx of heterotrophic C and N displayed a strong co-allocation for both elements in gastrodermis, epidermis, and host lipid bodies (Fig. 6B–D), substantially exceeding any photosynthate contribution in both host tissue layers. Especially for heterotrophic N, turnover in gastro- and epidermal compartment were one to two orders of magnitude higher compared to autotrophic input (Fig. 6B,C, Table S3).

Nutrients derived from the digestion of the isotopically enriched brine shrimps in the polyps were consistently detectable throughout the wider coenenchyme tissue after just 6 h as evidenced by similar levels of enrichment from randomly sampled coenenchyme patches in three independent coral colonies (cf. Fig. S4). NanoSIMS data revealed a strong gradient between host gastrodermis and epidermis with regard to C and N assimilation in both modes of nutrition (Fig. 6B,C), pointing towards a significant delay in the anabolic utilisation of nutrients between both layers or a fundamentally slower turnover in epidermal cells. The thin collagenous mesoglea appeared to act as a border that restricted flow between both tissue layers and limited the distribution and assimilation of nutrients primarily to the gastrodermal layer that contains the symbionts and faces the gastrovascular canal.

Both modes of nutrition displayed strikingly different patterns of nutrient allocation. Approximately 75% and 90% of the autotrophic C and N pool was present in the symbiont after 6 h (Fig. 7) and this strong retainment of nutrients, with a ~10 times higher N enrichment in symbiont compared to gastrodermis (Fig. 6A,B), also prevailed in a previous bulk study for the same coral after 12 h^56^. The remaining labelled material was mainly located in the gastrodermis and only 1–2% of the assimilated autotrophic nutrient pool was present in the epidermis, despite representing half of the area of the surface body wall coenenchyme in *Stylophora pistillata* (Fig. 7). In contrast, approximately two thirds of the heterotrophic nutrient pool was present in the gastrodermis under ambient conditions and a strong co-allocation of C and N with almost identical values for the partitioning of C and N across the three compartments was evident (Fig. 7). The remaining third was approximately equally split between symbiont and epidermal compartment, matching the relative compartment size in the case of the symbiont. In general, the epidermis was the main benefactor of the heterotrophic mode of nutrition and received a substantially larger share for both elements under ambient conditions compared to autotrophy (~22% *vs*. ~1%; Fig. 7).

The high congruence in heterotrophic C and N labelling in gastrodermis and different hotspots as well as their similar partitioning between compartments suggests that basic monomers containing both elements (e.g. amino acids) were used directly in anabolic processes, rather than being de-aminated or extensively processed involving internal (unlabelled) reserves. In contrast to the autotrophic mode of nutrition, heterotrophic C was not primarily directed towards host lipid bodies and turnover rates were four times lower compared to autotrophy (Fig. 6D). When extrapolated from these numbers, the single feeding event immediately (within 6 h) provided the host gastrodermis with the C and N equivalent of ~14 and ~283 daylight hours and epidermis with ~97 and ~903 daylight hours, respectively, of assimilated photosynthates. Although an earlier model calculation estimated that only one third of the daily N demand is covered through nitrate assimilation in *Stylophora pistillata*^3^, the heterotrophic N influx measured here would still considerably exceed the autotrophic N input (NO_3_^−^ and NH_4_^+^ make up 75% of the daily budget)^3^.

### Assimilation of heterotrophic nutrients by Symbiodinium

Heterotrophic N assimilation by the symbiont exceeded its own nitrate assimilation by a factor of two to three after 6 h, while C incorporation was approximately three times lower than the autotrophic input (Fig. 6A). The labelling of starch deposits in the symbiont with heterotrophic ^13^C (Fig. 2) demonstrates that coral catabolism released some brine shrimp C as CO_2_, likely through general decarboxylation reactions as well as the tricarboxylic acid (TCA) cycle. Indeed, comparing the fate of labelled C from incubation with 1 mM [1-^13^C]-pyruvate (label released as ^13^CO_2_ in the pyruvate dehydrogenase complex) or [3-^13^C]-pyruvate (^13^C-label enters TCA cycle) over 3 h in a separate experiment, confirmed that the initial decarboxylation of pyruvate, prior to entering the TCA cycle, is a strong contributor to symbiont C recycling (Fig. S4A,B). In contrast, fewer ^13^C seems to be photoassimilated from the CO_2_- release of the TCA cycle (Fig. S4D,E). The homogenous ^13^C labelling of host tissue and lipid bodies from [3-^13^C]-pyruvate (Fig. S4E) suggests that the label entered pathways of amino acid and fatty acid synthesis via cataplerotic reactions of the TCA cycle^60^ and/or direct through direct Acetyl-CoA use for fatty acid synthesis^61^. The strikingly different labelling pattern from these two pyruvate forms confirms the fixation of catabolic CO_2_ by the symbiont and the preferential allocation of translocated phototrophic carbon into the host lipid bodies. The degree to which host lipid bodies receive phototrophic carbon directly through the use of translocated symbiont-derived lipids or indirectly through sugar breakdown and *de novo* fatty acid synthesis via glycolytically derived Acetyl- and Malonyl-CoA by the host remains, however, to be established.

Substantial enrichment with heterotrophic N and the occurrence of ^15^N hotspots illustrated a rapid assimilation of brine shrimp N (Fig. 2). Our estimates that ~14–23% of the assimilated heterotrophic C and N pool was found in *Symbiodinium* after 6 h (based on cross-sectional areal budgets, Fig. 7), roughly match earlier observations in *Oculina arbuscula* and *O*. *diffusa* (10–20% of ^15^N after 4–28 h)^34,62^. Our data seem to support the notion of direct assimilation of prey N by the symbiont^34,62^, considering that the relative amount of replaced N in the symbiont population was on average only ~20% lower than in the surrounding host gastrodermis (Fig. 6A,B, Table S4). Piniak and Lipschultz^62^ argued that the presence of heterotrophic N in the symbiont within 4 h was insufficient time for a complete recycling of ^15^N from host metabolism (“i.e. host digestion, synthesis into host macromolecules, catabolism, excretion, uptake by zooxanthellae”). Their intracellular location and consistent assimilation of organic substrates (and amino acids in particular)^63–66^, would permit direct assimilation of amino acid monomers and released ammonium as they appear in the breakdown of the brine shrimp material (assuming transport across the symbiosome membrane). Indeed, the labelling pattern in NanoSIMS images from auto- and heterotrophically ^15^N-enriched symbiont cells was qualitatively similar (compare Figs 1C,F and S2B). In our interpretation, the small ^15^N hotspots in the symbionts represent the immediate concentration and storage of nitrate and ammonium ions into crystalline uric acid^40,46^, whereas the more or less homogenous labelling of all subcellular symbiont structures represents the incorporation of more complex N-containing compounds into the general cellular infrastructure. Despite the supplementing prey N, internal N recycling by the symbionts is a key mechanism for the coral and previous studies have estimated that up to ~80–90% of the symbiont N is derived from this process^18,35^.

### Elevated temperature reduces nutrient input and alters partitioning between tissue compartments

Elevated temperature consistently reduced nutrient assimilation in all compartments for both modes of nutrition (Fig. 6, Tables 1, S3 and S5) with the exception of autotrophic N assimilation in the host lipid bodies (Fig. 6D, Table S3). Structural incorporation of autotrophic C and N at elevated temperature declined on average by −19 ± 12% and −5 ± 26% in the symbiont, irrespective of feeding state (mean ± SD, N = 6; Fig. 6A). Likewise, autotrophic gastrodermal C and N incorporation declined significantly (−40 ± 29% and −29 ± 36%; Fig. 6B), with epidermal N turnover dropping on average by −33 ± 16% (Fig. 6C). For host lipid bodies, elevated temperature only significantly affected autotrophic C assimilation (−12 ± 17%; Fig. 6D). As previously reported, negative effects of prolonged elevated temperature (11.2 DHW) in this Red Sea *Stylophora pistillata* were largely restricted to lowered autotrophic nutrient turnover, but did not result in bleaching^42^. This observation also applies to the heterotrophic mode of nutrition. While still providing a net influx of nutrients, assimilation rates dropped generally by 45–70%. Temperature-induced reductions for heterotrophic N (all compartments) and C assimilation (gastrodermis, epidermis) were significantly larger than for the autotrophic mode (Fig. 6, Tables 1 and S3) and even fell to the level of autotrophic ambient temperature rates in two compartments (C in gastrodermis and N in symbiont; Fig. 6B,E). Concomitantly, heterotrophic hotspots also displayed significantly reduced average C- and N- enrichments (Fig. 6E). A previous study on the same Red Sea species has shown a reduced feeding rate at elevated temperature^67^. The decline in polyp ingestion rates of ~67% at 31 °C^67^ roughly matches our observed drop in average C and N assimilation (−57–66% for both host tissue layers) and would be consistent with the reduced enrichment in the ultrastructural features involved in food processing.

**Table 1:** The effect of temperature on autotrophic and heterotrophic input.

	Aut [F] *vs*. Het [F]	
	^13^C	^15^N
*Symbiodinium*	−22 ± 9% *vs*. −45 ± 44%	−4 ± 26% *vs*. −57 ± 37%*
host gastrodermis	−49 ± 27% *vs*. −66 ± 24%*	−22 ± 43% *vs*. −66 ± 24%*
host epidermis	−34 ± 35% *vs*. −57 ± 19%*	−29 ± 15% *vs*. −58 ± 18%*
host lipid bodies	−17 ± 12% *vs*. −55 ± 27%	0 ± 25% *vs*. −70 ± 18%*

Shown are the mean pairwise changes in autotrophic (Aut) and heterotrophic (Het) carbon and nitrogen turnover for each compartment of three tested colonies (mean ± SD) in regularly fed [F] corals (cf. Figs 6, S3). Data derived from the surface body wall of the coral coenenchyme. Pairs with asterisk indicate a significantly stronger reduction in heterotrophic than autotrophic turnover (Fig. 6, Table S3).

In addition to reducing overall C and N assimilation, elevated temperature caused a considerable shift in the partitioning of nutrients, especially for the heterotrophic mode of nutrition (Fig. 7). Here, the shift primarily benefited the symbiont and epidermal compartment and reduced the share of the gastrodermis from originally ~62% to ~48%. The consistent shift in the internal partitioning of nutrients for both modes of nutrition (albeit much more subtle in the autotrophic mode) that benefited symbiont and epidermis suggests a modulating impact of temperature specific to the gastrodermis as central hub of autotrophic and heterotrophic nutrient assimilation and distribution. We confirmed that the shift in heterotrophic nutrient partitioning at elevated temperature was truly related to nutrient allocation and assimilation efficiency and not linked to altered compartment sizes as e.g. observed in *Pocillopora damicornis*^68^.

Although the specific metabolic mechanism behind this shift in nutrient partitioning is yet unknown, our data illustrate that the acquisition of heterotrophic nutrients by the symbiont consist primarily of respiratory CO_2_ and organic and inorganic N derived from the food breakdown (see earlier discussion). Enhanced metabolic CO_2_ release might be indicative of a stronger diversion of heterotrophic food for catabolic activity on the host side. This would improve the energetic state of the host (i.e. ATP production) under elevated temperature, but at the same time enhance symbiont nutrient fixation and internal recycling. The hypothesis that enhanced gastrodermal catabolic activity benefits the symbiont at elevated temperature^69^ has so far not been possible to verify, because of the inability to directly measure proxies for respiratory activity on a tissue-specific scale. Increased catabolization of heterotrophic C is consistent with observations in some corals recovering from bleaching^70,71^. Assimilation of host catabolic CO_2_ (from prey and pyruvate) and the increased share of heterotrophic C and N in the symbiont at elevated temperature in our study provide new experimental data for the proposed role of heterotrophic feeding in alleviating symbiont nutrient and CO_2_-limitation at high temperatures^69^. To what degree host respiratory CO_2_ prevents sink limitation of photosynthesis and maintains autotrophic capacity under bleaching conditions requires, however, further experimental support.

## Conclusions

**Figure 8:**
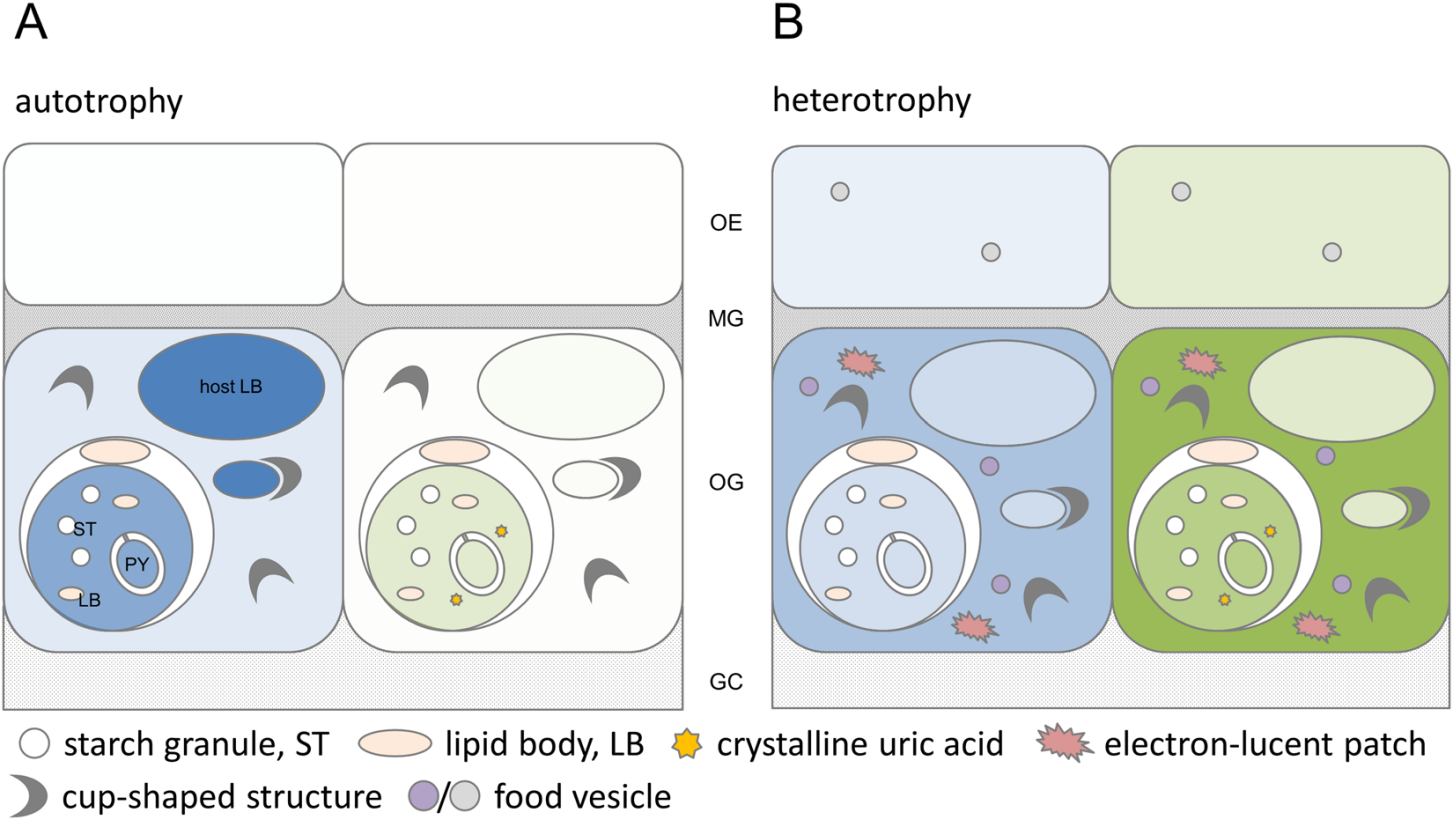
Schematic overview of nutrient input and ultrastructural features involved in the autotrophy and heterotrophy of *Stylophora pistillata*. Influx of carbon (left cell) and nitrogen (right cell) into symbiont, host lipid bodies, gastrodermal, and epidermal cell, originating from symbiont photoautotrophy (**A**; seawater bicarbonate and nitrate) or host heterotrophy (**B**; zooplanktonic prey) after 6 h. Colour tone contrasts the relative influx from both modes of nutrition for C (blue) and N (green), respectively. Subcellular structures involved in storage and processing of nutrients are highlighted (see text for details). OE, oral epidermis; MG, mesoglea; OG, oral gastrodermis; GC, gastrovascular canal.

By employing correlative TEM-NanoSIMS isotopic imaging, our study revealed the primary sites for the concentration and storage of nutrients derived from the two main fundamental modes of coral nutrition, auto- and heterotrophy. The microalgal partner derives C mainly from seawater DIC and N from internal recycling and heterotrophic input (Fig. 8). For the animal host, translocated photoautotrophic carbon is stored in lipid bodies, whereas heterotrophic C is processed involving multiple subcellular structures and primarily co-assimilated with N into the host tissue (Fig. 8).

We highlighted the critical role that mode of nutrition and seawater temperature have on the relative turnover and partitioning of C and N within the different tissues of the intact symbiosis. This study nevertheless represents a 6 h snapshot of the tissue in a healthy coral known to have a comparably high temperature threshold in its location^42^. Thus, the observed role of autotrophy and heterotrophy has to be considered within this context. A number of studies have shown the significant role that heterotrophy plays in the survival and recovery of corals after severe thermal stress events that left their autotrophic capacity impaired^70,72–76^. At the same time, as future oceans will be less alkaline, direct negative effects on coral feeding rates^77,78^ and/or indirect negative effects of ocean acidification on zooplankton abundance in reefs^79^ will create specific demands for the autotrophic and heterotrophic mode of nutrition in individual corals. While the preservation of symbiont functioning and initial host energy reserves are the foremost elements of coral resistance to thermal stress^80^, it is the complementing interplay of auto- and heterotrophy that defines the nutritional state and ability of corals to recover after a severe disturbance of their symbiotic state in a warmer ocean^81^.

## Methods

### Coral maintenance and experimental design

Specimens of *Stylophora pistillata* were collected from the shallow waters (4–8 m) in the Gulf of Aqaba in Eilat, Israel under permit 2013/40158 of the Israel Nature and Parks Authority. Experiments were conducted in the outdoor Red Sea Simulator system at the Interuniversity Institute (IUI) for Marine Sciences^82^ and corals maintained in flow-through aquaria (30 L; >60 L h^−1^) under ambient water temperature and reduced solar irradiance (ca. 350 µmol m^−2^ sec^−1^ at midday). Three different mother colonies were fragmented to provide six similarly sized (4–8 cm) clonal fragments per colony. Six clonal fragments from three different mother colonies each were acclimated to the aquarium settings for 3 weeks and then allocated to four treatments (ambient *vs*. elevated temperature with or without feeding) in a paired design and maintained over 67 days (Fig. S1). The heat-treated fragments (originating from colonies A, G, I) underwent a thermal profile with a cumulative heat exposure of 11.2 degree heating weeks (DHW) at ambient pH 8.1, identical to Krueger *et al*.^42^. Corals in the feeding treatments were fed twice a week with freshly hatched *Artemia* nauplii (2500 per coral fragment) for one hour. The daily mean temperature in the ambient and elevated treatment for the ten days leading up to the experimental incubation was 24.1 ± 0.2 °C and 29.1 ± 0.2 °C, respectively^42^.

### Isotopic labelling of seawater for autotrophic pulse

Seawater for the autotrophic labelling pulse was spiked with 2 mM NaH^13^CO_3_ (98 atom %) and 3 µM K^15^NO_3_ (98 atom %). The chosen 3 µM nitrate spike for the 6 h labelling period brings the total nitrate concentration to approximately ten times higher than natural values for this location (0.2–0.3 µM) and most other reef locations^83,84^, but nevertheless represents a nutrient level that does naturally occur in some reef locations, e.g. near equatorial upwelling regions (e.g. Galapagos islands; 3–5 µM), in nearshore regions of the Great Barrier Reef and Coral Triangle (~1–3 µM), and in some reefs in the Gulf region (e.g. Strait of Hormuz, Gulf of Oman; 2–3 µM)^84–86^.

### Isotopic labelling of Artemia sp. for heterotrophic pulse

*Tetraselmis* sp. cultures that would later serve as food source to label *Artemia* sp. were grown in modified f/2-medium (-Si), supplemented with 2 mM NaH^13^CO_3_ and Na^15^NO_3_ (0.882 mM final concentration) as semi-continuous culture for 3 weeks. Final enrichment of a lyophilized aliquot was 69.58 atom % for ^13^C and 40.23 atom % for ^15^N. *Artemia* cultures were fed daily with this *Tetraselmis* culture for nine days after hatching in order to maximize enrichment, while avoiding that *Artemia* individuals became too large to serve as life prey for *Stylophora pistillata*. *Artemia* cultures were maintained in a 30 L outdoor aquarium tank that was also supplemented with 2 mM NaH^13^CO_3_ and 3 µM Na^15^NO_3_ to minimize loss of labelling in remaining *Tetraselmis* cells and label other phytoplankton that potentially grew in this feeding tank. At the end of the labelling period all *Artemia* individuals were collected with a plankton net and concentrated in 2.5 L to assess the size of the population and determine the feeding density for the experiment. A bulk *Artemia* subsample that underwent the same resin embedment as the coral samples was measured using NanoSIMS and the mean ^13^C and ^15^N enrichments were 4.77 atom % and 33.77 atom %, respectively. The purpose of this was to normalize the measured enrichment in the coral sample to the enrichments in seawater and prey to adequately compare both modes of nutrition (see section Data treatment and normalisation).

### Experimental incubations

Incubations with labelled seawater or labelled food were done in 30 L aquaria and in- and outflow was interrupted for the labelling period with water movement solely provided by the heat exchanger and the surface pump. To assess whether the autotrophic assimilation is affected by regular host feeding, one set of corals that had been acclimated to regular feeding in both temperatures was moved into the aquaria used for the autotrophic labelling pulse one day before the experiment. Thus, three different incubation modes were performed: autotrophic pulse with unfed corals (Aut [U]), autotrophic pulse with regularly fed corals (Aut [F]), and heterotrophic pulse with regularly fed corals (Het [F]) (Fig. S5). Coral fragments in both temperature treatments were exposed to labelled seawater or one batch of labelled living *Artemia* (~1059 individuals per fragment at a density of 424 individuals L^−1^) for a total of 6 h (10 am to 4 pm local time). Corals were moved every two hours into new tanks to minimize the effects of increasing water temperature due to the high ambient air temperature. Density of labelled living *Artemia* in the water column over time was monitored by counting individuals from 30 mL water samples in a zooplankton counting chamber (Hydrobios Apparatebau GmbH, Altenholz, Germany) under a binocular every 20 mins. After adding *Artemia* prey to the aquarium, corals developed mucus threads and many *Artemia* got visibly trapped or directly captured by coral polyps; there were no individuals in the water column after 80 mins.

### Sample preparation, TEM, and NanoSIMS

At the end of the isotopic pulse, the apical tip of the coral branch was removed and discarded and a ~1 cm coral piece was clipped off and fixed (2.5% [v/v] glutaraldehyde, 0.5% [v/v] formaldehyde in 0.22 µm filtered 0.1 M phosphate buffer with 0.6 M sucrose, pH 7.4–7.6) for 2 h at room temperature, followed by 22 h at 4 °C. A piece of an unlabelled coral colony from fed and unfed conditions was used as reference to assess the natural isotopic ratio in the tissue of interest. Pieces were completely decalcified in 0.5 M EDTA. Fixed tissue samples were dissected to obtain connective coenenchyme tissue and post-fixed in 1% [v/v] osmium tetroxide in water for 1 h. We exclusively analysed the coenenchyme tissue between polyps in order to avoid data artefacts due to the individual polyps catching different amounts of prey within the pulse period. All used histological terminology follows Peters^23^. Post-fixed samples were dehydrated, embedded in Spur resin blocks, sectioned and post-stained for TEM, following established protocols^9^. For correlative TEM and NanoSIMS, ultrathin sections (70 nm) were mounted on carbon formvar-coated Finder copper grids and TEM images obtained at 80 or 100 kV with a Philips CM 100 and a Tecnai 12 (FEI) TEM, respectively. In order to cover larger areas and to acquire sufficient data for statistical analysis, semi-thin sections (500 nm) were mounted on glass slides and directly imaged with NanoSIMS.

Suitable areas were analysed for ^13^C and ^15^N enrichment using a NanoSIMS 50 L ion microprobe. Sections were gold-coated and bombarded with a 16 keV primary Cs^+^ ion beam. Raster scans of the area of interests were performed (40 × 40 µm image size; 256 × 256 pixels with 5 ms dwell time per pixel; beam focus spot size of ~150 nm; 6 acquisition layers). The secondary ions ^12^C_2_^−^ (mass 24), ^13^C^12^C^−^ (mass 25), ^12^C^14^N^−^ (mass 26), and ^12^C^15^N^−^ (mass 27) where simultaneously counted in electron multipliers at a mass resolution of about 10000, enough to resolve all potential interferences. Images provided a complete cross section of the surface body wall epidermis and gastrodermis with sufficient resolution for subcellular features to be clearly resolved. Image sizes for correlative TEM and NanoSIMS varied. For semi-thin sections, approximately 10–15 images were obtained per replicate to obtain enrichment values for 40–60 symbiont cells and their surrounding host tissue. In case multiple sections from the same sample block were required, sections were taken at least 20 µm apart to avoid cutting and imaging another layer of the same symbiont cells.

### Data treatment and normalisation

NanoSIMS data were processed using the L’IMAGE software (created by Dr. Larry Nittler, Carnegie Institution of Washington). Maps of ^13^C/^12^C and ^15^N/^14^N ratio distributions were derived from drift-corrected secondary ion images. Correlative TEM and NanoSIMS images were used to identify enriched subcellular structures. Note that TEM images were adjusted for contrast. Obtained isotopic ratios (R_sample_) were converted into ^13^C and ^15^N atom fractions, *F* (in atom %, AP, when expressed as *F* × 100),

1 $${F}_{sample}=\,\frac{{R}_{sample}}{1+{R}_{sample}}$$

and expressed as atom percent excess (APE in %) above the standard fraction in an unlabelled sample^87^. Note that NanoSIMS ^13^C/^12^C ratios are twice the standard ratio (measured as C_2_) and were thus divided by the factor two for the following calculations.

Regions of interest (ROIs) were drawn on ratio images to assess average enrichment in each type of compartment for the following ROIs: Symbionts (cross-sectional diameter >3 µm), extra-algal lipid bodies (outside of symbionts, but contained within the symbiosome), host gastrodermis (excluding symbionts), host epidermis, and host lipid bodies (diameter >1 µm). In addition, isotopic hotspots in the heterotrophic treatments were measured. Each NanoSIMS image thus provided data points for multiple symbiont and lipid ROIs and one data point for gastrodermis and epidermis, respectively. All NanoSIMS images were smoothed (smooth width of 3 pixels).

In order to accurately contrast the input from autotrophic and heterotrophic nutrients, the measured enrichment in each ROI was normalized to the isotopic labelling of the respective source (seawater or brine shrimp). The atom ratios for the resin embedded heterotrophic food source were determined with *F*_Het_C_ = 0.04772 ± 0.01155 and *F*_Het_N_ = 0.33772 ± 0.01360 (N = 6). Atom fractions of ^13^C and ^15^N in the spiked seawater (*F*_SW_C_ and *F*_SW_N_) were calculated. Using the standard atom fractions for C (*F*_PDB_: 0.01111) and N (*F*_N_: 0.00366)^87^ in the natural seawater and ambient concentrations of 2.2 mM total dissolved inorganic C and 0.3 µM nitrate^83^, the following equations express the final seawater atom fractions in 1 L of seawater after the addition of the spike (*F*_SW_); with n in mmol and *F* as atom fraction. Atom fractions for the added spike were 0.98 for both NaH^13^CO_3_ and K^15^NO_3_, as indicated by the supplier (Sigma-Aldrich, St Louis, MO, USA).

2 $${F}_{S{W}_{C}}=\frac{({n}_{SWDIC}\times {F}_{PDB})+({n}_{SPIKEDIC}\times {F}_{SPIKEDIC})}{{n}_{SWDIC}+{n}_{SPIKEDIC}}=\frac{(2.2\times 0.01111)+(2.0\times 0.98)}{2.2+2.0}=0.4725$$

3 $${F}_{S{W}_{N}}=\frac{({n}_{SWnitrate}\times {F}_{nitrogen})+({n}_{SPIKEnitrate}\times {F}_{SPIKEnitrate})}{{n}_{DICnitrate}+{n}_{SPIKEnitrate}}=\frac{(0.0003\times 0.00366)+(0.003\times 0.98)}{0.0003+0.003}=0.8912$$

Using these *F*_SW_ values, the normalized APE in autotrophic samples was calculated for each element as:

4 $$AP{E}_{autotrophy}[ \% ]=\frac{{F}_{sample}-{F}_{reference}}{{F}_{S{W}_{C\mathrm{or}N}}-{F}_{PDB\mathrm{or}nitrogen}}\times 100$$

Using the measured atom fraction from labelled and unlabelled *Artemia* in resin, the normalized APE for the heterotrophic coral samples was calculated as:

5 $$AP{E}_{heterotrophy}[ \% ]=\frac{{F}_{sample}-{F}_{reference}}{{F}_{Artemia}-{F}_{reference}}\times 100$$

In essence, these normalized APE for each type of ROI express the proportion of the total C or N pool that has been replaced with newly assimilated C and N from either food source. Assuming a negligible change in total biomass in the imaged coenenchyme areas within the pulse period (steady state assumption of C and N pool), APE is a measure of the relative C and N turnover in each ROI over 6 h in the light. Note that conventional preparation of biological samples for NanoSIMS involves a loss of most soluble compounds and restricts observations mainly to the anabolic part of metabolism (the building of cellular structures). Thus, the presented values should be interpreted as minimal estimates for tissue C and N assimilation.

Since APE only represents the relative turnover of a compartment without considering the actual size of this compartment, a semi-quantitative measure of C and N assimilation was calculated by multiplying size of compartment (cross-sectional area A, in µm^2^) with relative turnover in this compartment (APE in %) for each element. This “integrated area fraction” (IAF) for each type of compartment allows an assessment of relative C and N partitioning across the main three tissue compartments of the surface body wall within the 6 h pulse, where the total assimilated C or N pool is composed of:

6 $$\mathrm{total}\,\mathrm{pool}\,\mathrm{size}=(IA{F}_{symbiont})+(IA{F}_{gastrodermis})+(IA{F}_{epidermis})$$

Note, that IAF is a semi-quantitative measure based on the reasonable assumption that the C/N elemental ratio in these compartments is similar, consistent with NanoSIMS images (data not shown). For a fully quantitative comparison, one would have to determine the total C- and N-pool in each compartment separately. Note that IAFs and the resulting partitioning budget only considers the structurally incorporated C and N; i.e. it does not consider losses due to mucus shedding or respiration.

### Statistical analysis

Considering that ROI enrichment values contain two sources of variability, resulting from the cutting level within the cell (affecting for example the abundance of starch granules in a particular symbiont ROI) and the true biological variability as result of size, age and physiological state of individual cells, we decided to treat the ROI data points as raw data points and include biological replicate as a factor in the analysis, rather than treating multiple ROIs from one biological replicate as technical replicates and reducing them to a single value. Thus, we sought to balance the number of data points for the same types of ROI between the three biological replicates for statistical considerations. Note that a previously published unfed autotrophy dataset^42^ was reused here and contrasted with other feeding conditions. Depending on the specific question, the three datasets (Aut [U], Aut [F], Het [F]) were tested individually or jointly with multifactorial ANOVAs for each ROI type. Data were transformed where necessary to meet assumptions of normality (Shapiro-Wilk test). Homogeneity of variance was tested via Levene’s test. All models were reduced to minimal adequate models and significant interaction terms analysed with Tukey HSD *post hoc* tests. The following tests were conducted: (i) whether regular host feeding affects the autotrophic assimilation at different temperatures within each compartment was tested with a Three-Way ANOVA with temperature, feeding acclimation, and replicate as factors (Aut [U] *vs*. Aut [F]; Table S2); (ii) the heterotrophy dataset (Het [F]) was tested for the effect of temperature (Table S5); (iii) the difference in the tissue-specific C and N contribution from autotrophy and heterotrophy and how elevated temperature affects this was tested with a Three-way ANOVA for each compartment, with temperature, mode of nutrition and replicate as factors (Aut [F] *vs*. Het [F]; Table S3).

## Electronic supplementary material

Supplementary Figures and Tables

## Data Availability

Summary NanoSIMS data for each ROI category by mode of nutrition, feeding acclimation, temperature, and replicate are provided as Table S4. All images generated and/or analysed in the current study are available from the corresponding author on reasonable request.
