# Supplementary material for: Temperature and feeding induce tissue level changes in autotrophic and heterotrophic nutrient allocation in the coral symbiosis – A NanoSIMS study
Source: Sci Rep. 2018 Aug 23;8:12710. doi: 10.1038/s41598-018-31094-1 (PMC6107511; doi:10.1038/s41598-018-31094-1)
Supplement: Supplementary file 1 — Supplementary Figures and Tables [file 41598_2018_31094_MOESM1_ESM.pdf]

Temperature and feeding induce tissue level changes in autotrophic and heterotrophic nutrient allocation in the coral symbiosis – A NanoSIMS study

Thomas Krueger, Julia Bodin, Noa Horwitz, Céline Loussert-Fonta, Adrian Sakr, Stéphane Escrig, Maoz Fine, Anders Meibom

Supplementary Information

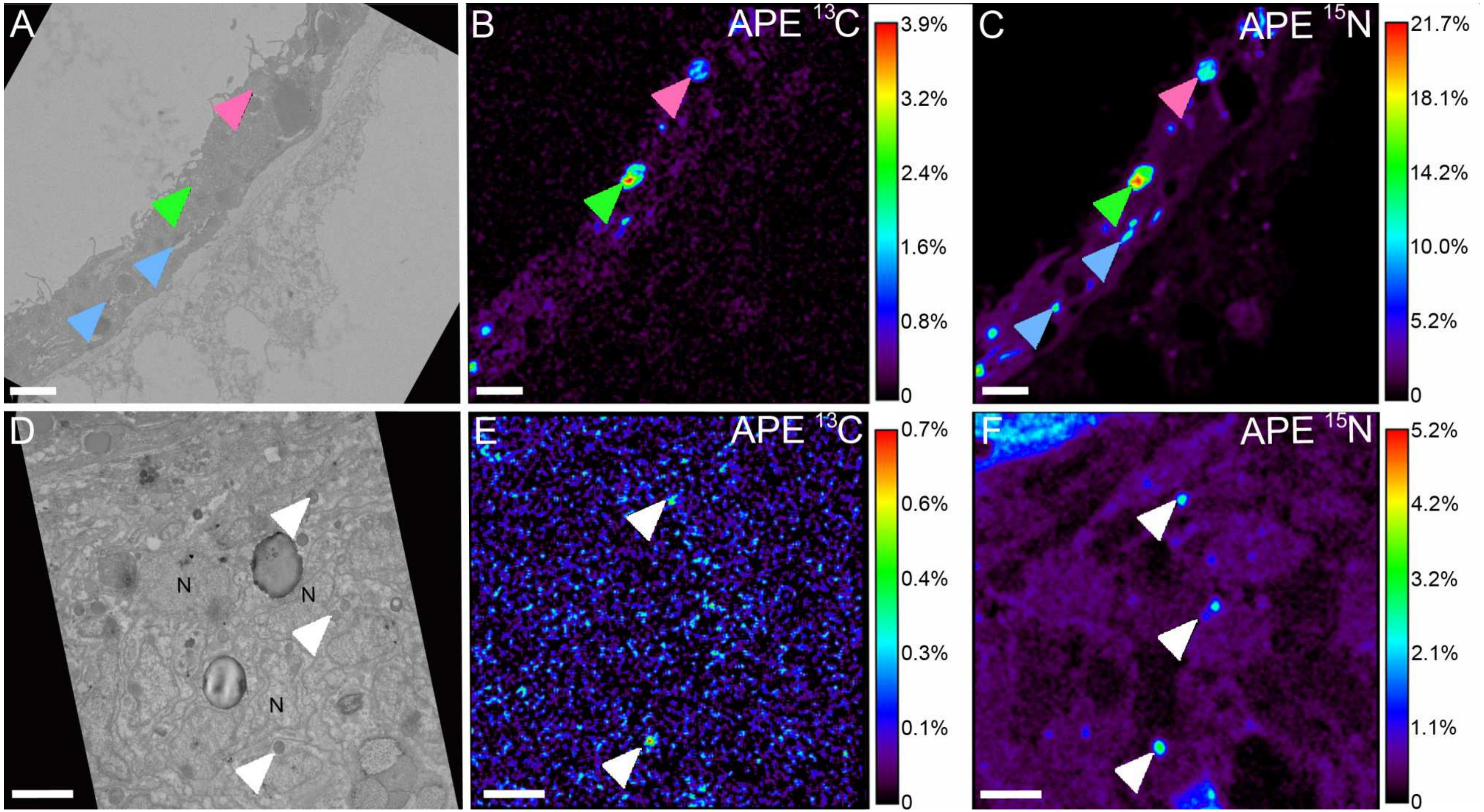

Figure S1. Heterotrophic carbon and nitrogen allocation in the basal body wall gastrodermis and surface body wall epidermis. Representative images for the distribution of prey ^{13}C and ^{15}N in the coral basal body wall gastrodermis (A-C) and the surface body wall epidermis (D-F), originating from ingestion of labelled brine shrimps after 6h. Colour codes of arrows in A-C correspond to the three described types of gastrodermal hotspots (Fig. 4). White arrows in D-F indicate multivesicular bodies. Colours in NanoSIMS maps display enrichment relative to an unlabelled tissue in δ-notation (black to red). GC: gastrovascular canal, CD: calicodermis, N: nucleus. Scale bars are 2 µm.

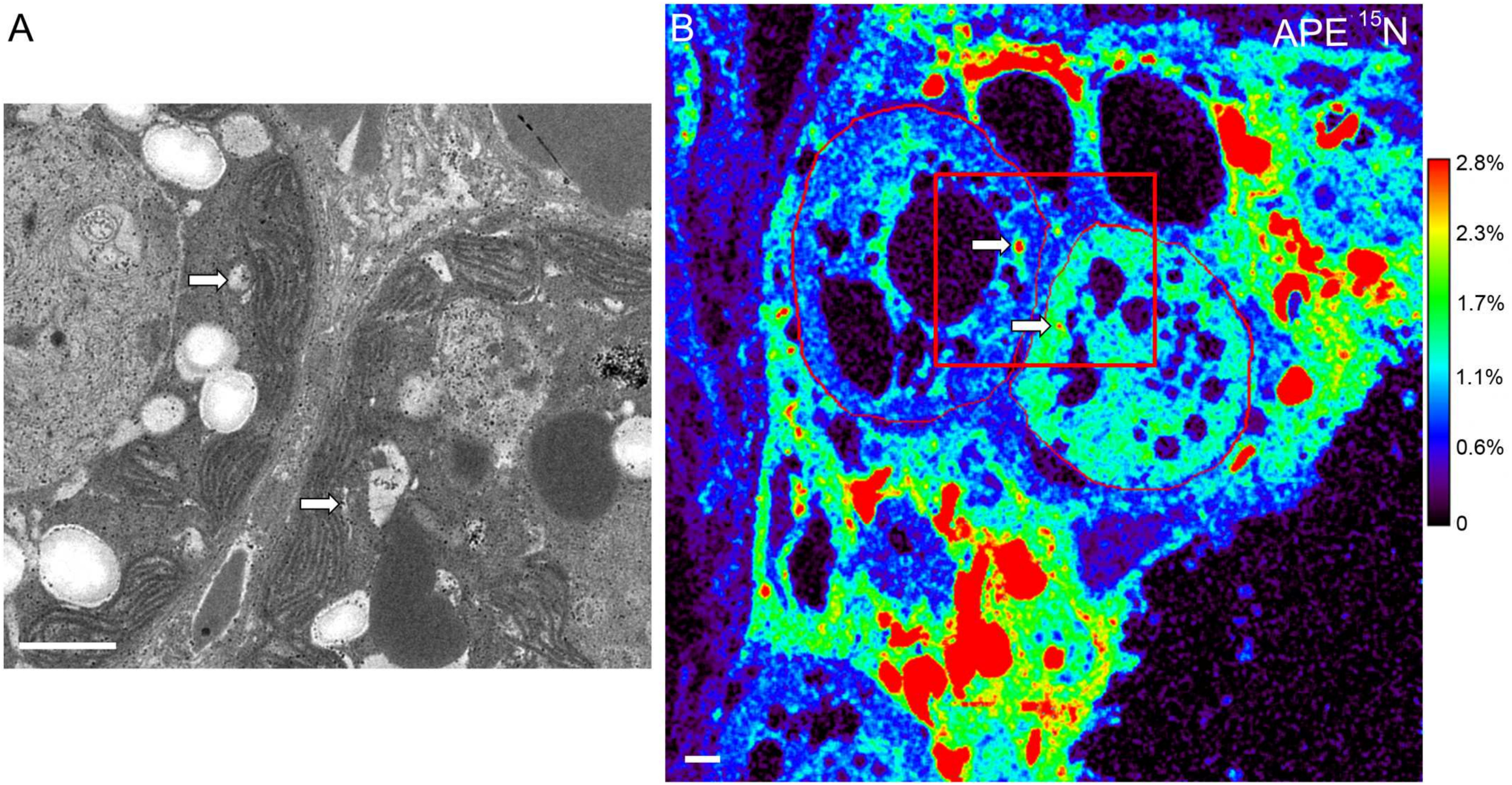

Figure S2. Heterotrophic nitrogen hotspots in *Symbiodinium*. TEM (A) and corresponding ^{15}N NanoSIMS image (B) reveals homogenous heterotrophic nitrogen labelling in the symbiont cells (thin red outline) with occasional vesicle-like hotspots (white arrows). Note that (B) shows the same image as Fig. 2F with an APE-value cut-off at 2.8%. Scale bars are 1 µm.

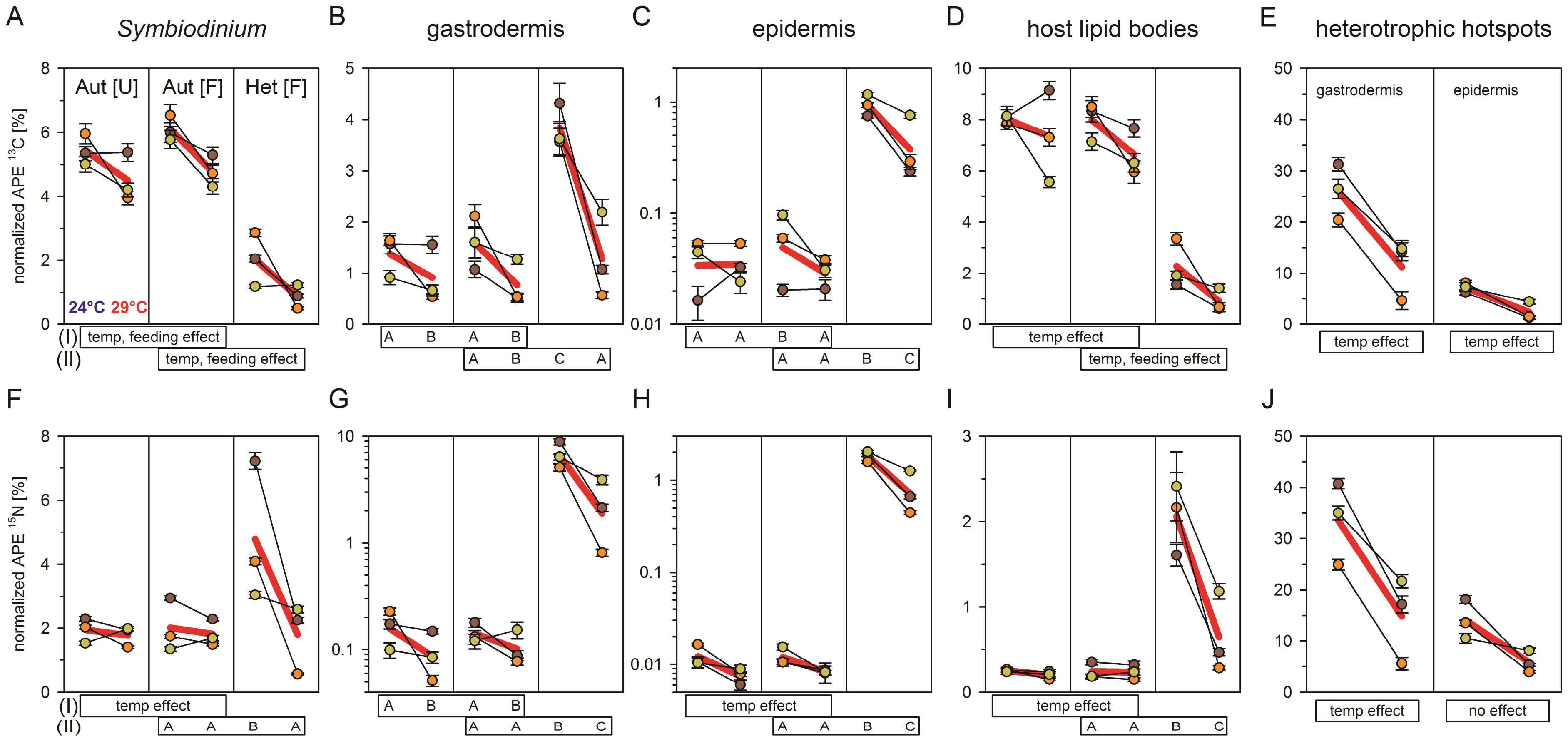

Figure S3. Colony-specific data for compartment-specific carbon and nitrogen turnover. Normalized carbon (A-E) and nitrogen (F-J) turnover for all coral compartments, showing individual colony responses (dots; green, orange, red; mean±SE) and overall species response (thick red line) identical to data shown in Fig. 6. Statistical effects of (I) temperature and feeding acclimation in Aut [U] *vs*. Aut[F] (cf. Table S2) and of (II) temperature and mode of nutrition in Aut [F] *vs*. Het [F] (cf. Table S3) on C and N turnover are shown below each subpanel. In case of significant interactions of the two main factors, Tukey HSD *post hoc* results are depicted as capital letters, where treatments not connected by the same letter are significantly different.

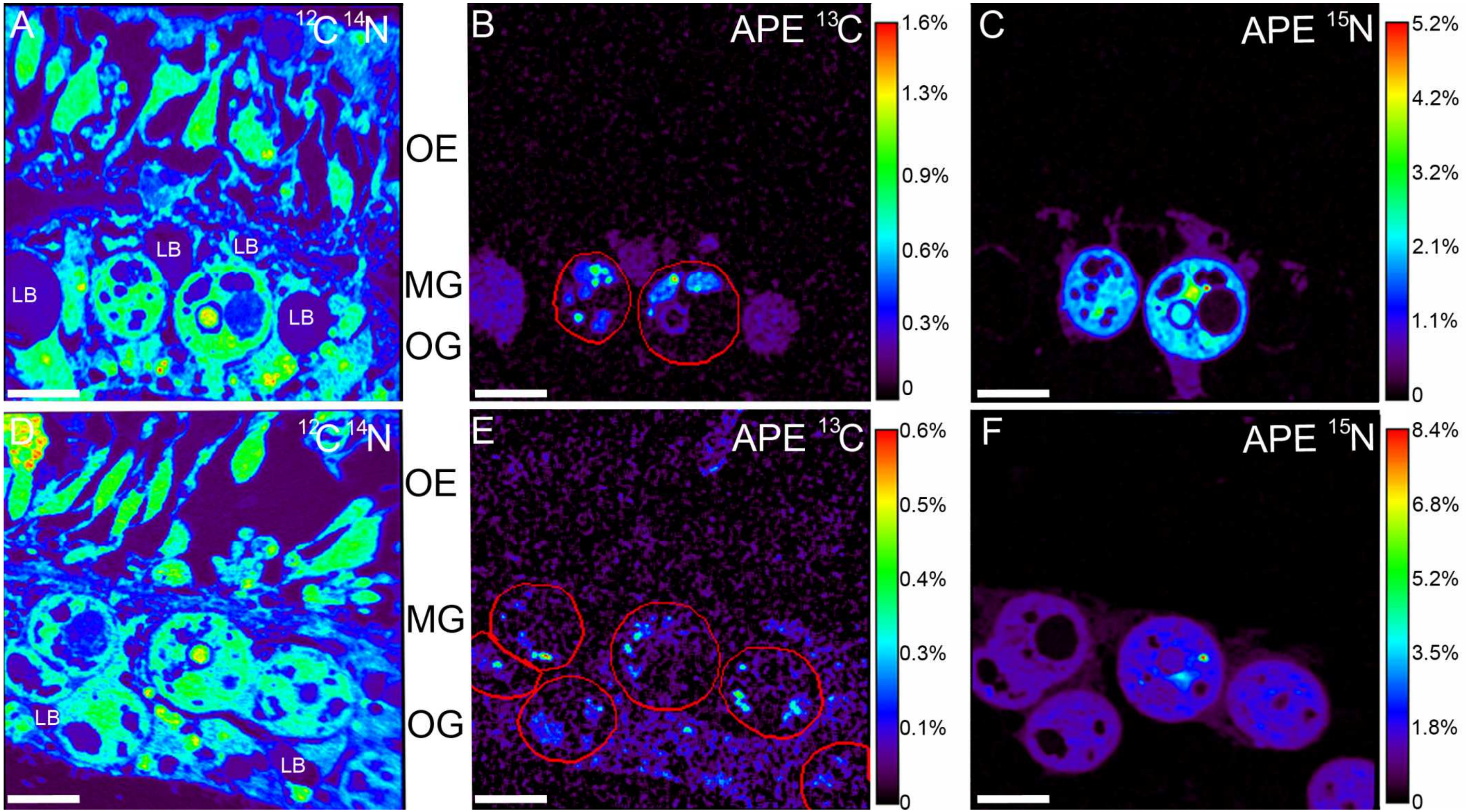

Figure S4. The fate of pyruvate carbon. Enrichment patterns of coenenchyme tissue of *Stylophora pistillata* after incubation with 1 mM [1-^{13}C]-pyruvate + 3 µM $K^{15}NO_3$ (A-C) and [3-^{13}C]-pyruvate + 3 µM $K^{15}NO_3$ (D-E) after 3h in the light. General tissue structure (A, B) showing oral epidermis (OE), mesoglea (MG), and oral gastrodermis (OG), containing symbionts and host lipid bodies (LB). Average symbiont APE of shown symbiont cells was 0.168% *vs*. 0.039% (B *vs*. E) and 1.78% *vs*. 1.29% (C *vs*. F). Scale bars are 5 µm.

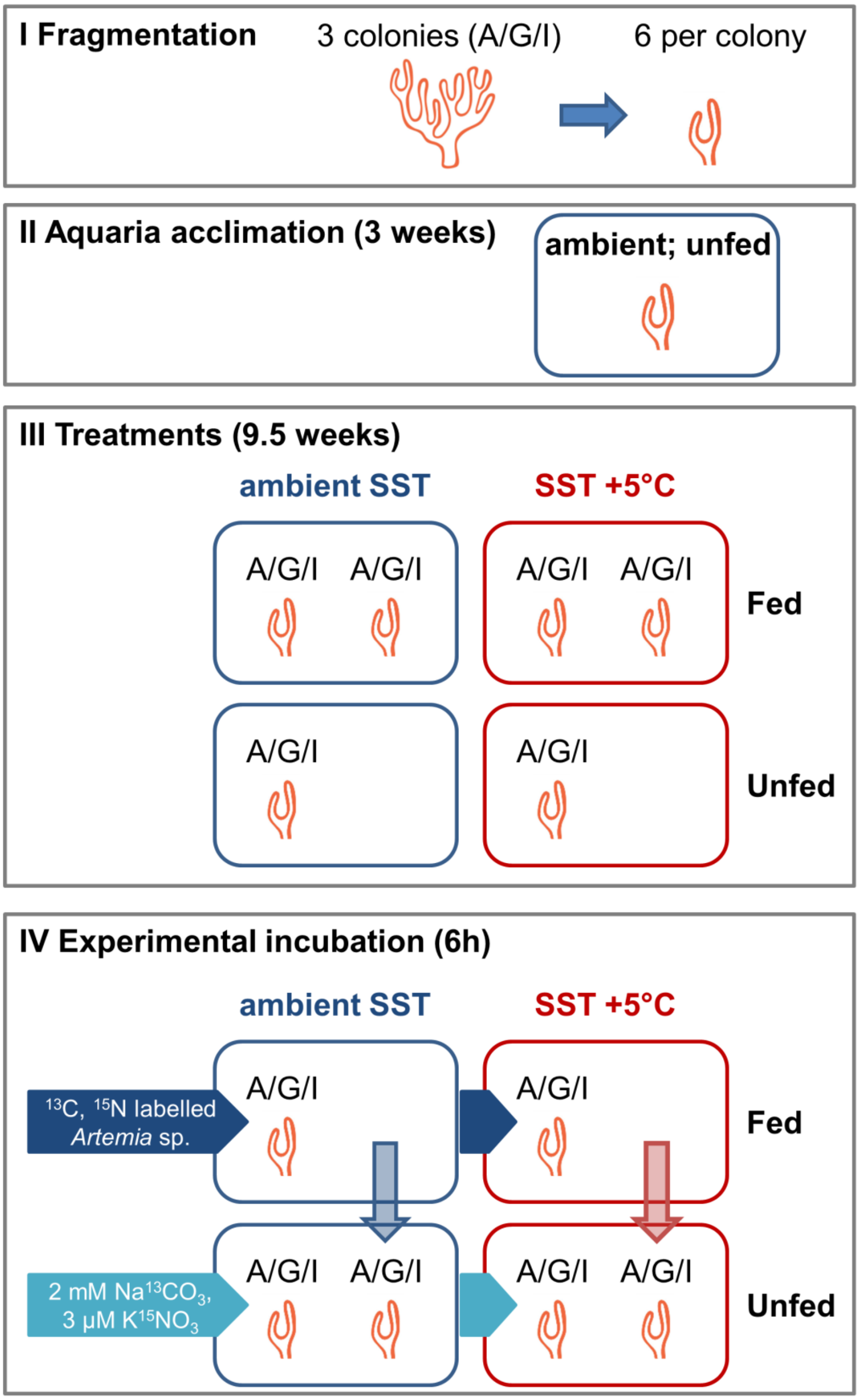

Figure S5. Schematic overview of experimental setup and coral acclimation. After collection from the sea, three coral colonies (A/G/I) were fragmented (I) and acclimated to the aquarium setup (II). Corals were subsequently maintained under a 2x2 factorial treatment design with 2 levels for temperature (ambient SST/SST+5°C) and food supply (Fed/Unfed) (III) in a paired manner (all individuals went into all treatments). For the experimental incubation (IV), the isotopic label was either provided in the form of inorganic nutrients to assess autotrophic assimilation or as organic prey (*Artemia salina*) for heterotrophic assimilation. To contrast autotrophic performance in corals that are acclimated to a regular food input *vs*. corals that are fully dependent on autotrophy, fed coral fragments from each temperature treatment were moved into the aquaria that received inorganic isotopic labels.

Table S1. Statistical effects of feeding and temperature on selected physiological variables of the *Stylophora pistillata* holobiont in colonies A, G, I, based on mixed model analysis of a paired factorial design. Asterisks indicate statistical significance with arrows indicating the direction of the effect.

variable	feeding	temperature	feeding x temperature	REML variance component estimate
host protein [mg cm^{-2}]	$F_{1,6}$ = 4.002 p=0.0924	$F_{1,6}$ = 0.699 p=0.4350	$F_{1,6}$ = 0.0355 p=0.8568	10%
host carbohydrates [µmol cm^{-2}]	$F_{1,6}$ = 0.458 p=0.5240	$F_{1,6}$ = 0.327 p=0.5882	$F_{1,6}$ = 0.231 p=0.6480	9%
symbiont protein [pg $cell^{-1}$]	$F_{1,6}$ = 4.056 p=0.0906	$F_{1,6}$ = 1.973 p=0.2097	$F_{1,6}$ = 0.386 p=0.5575	75%
symbiont carbohydrate [pg $cell^{-1}$]	$F_{1,6}$ = 0.017 p=0.8995	$F_{1,6}$ = 2.195 p=0.1890	$F_{1,6}$ = 0.279 p=0.6165	0%
symbiont density [cells cm^{-2}]	$F_{1,6}$ = 5.797 p=0.0527	$F_{1,6}$ = 1.331 p=0.2926	$F_{1,6}$ = 0.4645 p=0.5209	82%
symbiont density [cells mg host $protein^{-1}$]	$F_{1,6}$ = 0.008 p=0.9304	$F_{1,6}$ = 3.133 p=0.1271	$F_{1,6}$ = 0.396 p=0.5523	59%
total Chl content [pg $cell^{-1}$]	$F_{1,6}$ = 6.268 p=0.0463* ↑	$F_{1,6}$ = 20.487 p=0.0040* ↑	$F_{1,6}$ = 4.166 p=0.0873	0%
O_2 per Chl [mg min^{-1} µg Chl^{-1}]	$F_{1,6}$ = 8.491 p=0.0268* ↓	$F_{1,6}$ = 0.490 p=0.5102	$F_{1,6}$ = 3.857 p=0.0972	22%
O_2 per surface [mg min^{-1} cm^{-2}]	$F_{1,6}$ = 0.244 p=0.6392	$F_{1,6}$ = 9.374 p=0.0222* ↑	$F_{1,6}$ = 0.570 p=0.4788	6%
respiration [mg O_2 min^{-1} mg $protein^{-1}$]	$F_{1,6}$ = 0.590 p=0.4715	$F_{1,6}$ = 0.057 p=0.8190	$F_{1,6}$ = 0.083 p=0.7832	9%
respiration [mg O_2 min^{-1} cm^{-2}]	$F_{1,6}$ = 0.188 p=0.6795	$F_{1,6}$ = 0.069 p=0.8019	$F_{1,6}$ = 0.057 p=0.8191	0%
P_{gross}:R	$F_{1,6}$ = 2.154 p=0.1926	$F_{1,6}$ = 21.079 p=0.0037* ↑	$F_{1,6}$ = 0.562 p=0.4819	50%

Table S2. Statistical results of the Three-Way ANOVA on the effects of feeding acclimation, temperature, and replicate on the autotrophic fixation of bicarbonate and nitrate for each region of interest (cf. Fig. 6 Aut [U] *vs*. Aut [F]). Note that ^{15}N ROI data was not considered for the extra-algal lipid bodies due to their close proximity to the highly enriched symbiont cell and the labelled symbiosome membrane (cf. LB[*] in Fig. 1C). In contrast ^{13}C enrichment was clearly assessable (Fig 1B). Asterisks indicate statistical significance.

**element**	**compartment**	***Symbiodinium***	**gastrodermis**	**epidermis**	**host lipid bodies**	**extra-algal lipid bodies**
^{13}C	feeding acclimation (F)	$F_{1,607}$ = 9.893, p=0.0017*	$F_{1,158}$ = 0.187, p=0.6663	$F_{1,158}$ = 6.751, p=0.0103*	$F_{1,522}$ = 2.532, p=0.1122	$F_{1,129}$ = 0.203, p=0.6534
	temperature acclimation (T)	$F_{1,607}$ = 54.047, p<0.0001*	$F_{1,158}$ = 69.107, p<0.0001*	$F_{1,158}$ = 27.203, p<0.0001*	$F_{1,522}$ = 24.527, p<0.0001*	$F_{1,129}$ = 2.677, p=0.1044
	replicate (R)	$F_{2,606}$ = 7.307, p=0.0007*	$F_{2,157}$ = 0.431, p=0.6508	$F_{2,157}$ = 46.836, p<0.0001*	$F_{2,521}$ = 19.674, p<0.0001*	$F_{2,128}$ = 1.787, p=0.1718
	F x T	N/A	$F_{1,156}$ = 5.877, p=0.0165*	$F_{1,156}$ = 22.731, p<0.0001*	$F_{1,520}$ = 2.956, p=0.0862	N/A
	T x R	$F_{2,603}$ = 8.552, p=0.0002*	$F_{2,154}$ = 18.585, p<0.0001*	$F_{2,154}$ = 27.344, p<0.0001*	$F_{2,518}$ = 8.519, p=0.0002*	$F_{2,125}$ = 3.560, p=0.0314*
	F x R	N/A	$F_{2,154}$ = 30.607, p<0.0001*	$F_{2,154}$ = 14.204, p<0.0001*	$F_{2,518}$ = 0.717, p=0.4889	$F_{2,125}$ = 3.448, p=0.0349*
	F x T x R	N/A	N/A	N/A	$F_{2,512}$ = 10.200, p<0.0001*	N/A
^{15}N	feeding acclimation (F)	$F_{1,607}$ = 1.890, p=0.1697	$F_{1,158}$ = 0.548, p=0.4602	$F_{1,158}$ = 0.158, p=0.6913	$F_{1,522}$ = 0.003, p=0.9558	-
	temperature acclimation (T)	$F_{1,607}$ = 22.872, p<0.0001*	$F_{1,158}$ = 45.346, p<0.0001*	$F_{1,158}$ = 58.990, p<0.0001*	$F_{1,522}$ = 4.764, p=0.0295*	-
	replicate (R)	$F_{2,606}$ = 151.771, p<0.0001*	$F_{2,157}$ = 6.219, p=0.0025*	$F_{2,157}$ = 5.934, p=0.0033*	$F_{2,521}$ = 14.396, p<0.0001*	-
	F x T	$F_{1,605}$ = 0.054, p=0.8159	$F_{1,156}$ = 4.541, p=0.0347*	$F_{1,156}$ = 0.789, p=0.3760	N/A	-
	T x R	$F_{2,603}$ = 60.557, p<0.0001*	$F_{2,154}$ = 18.570, p<0.0001*	$F_{2,154}$ = 0.8326, p=0.4370	N/A	-
	F x R	$F_{2,603}$ = 34.306, p<0.0001*	$F_{2,154}$ = 9.4011, p<0.0001*	$F_{2,154}$ = 6.149, p=0.0027*	$F_{2,518}$ = 6.182, p=0.0022*	-
	F x T x R	$F_{2,597}$ = 6.777, p=0.0012*	$F_{2,148}$ = 12.173, p<0.0001*	$F_{2,148}$ = 9.474, p=0.0001*	N/A	-

Table S3. Statistical results of the Three-Way ANOVA on the effects of mode of nutrition (autotrophic *vs*. heterotrophic), temperature, and replicate on the carbon and nitrogen turnover for each region of interest (cf. Fig. 6: Aut [F] vs. Het [F]). Asterisks indicate statistical significance.

**element**	**compartment**	***Symbiodinium***	**gastrodermis**	**epidermis**	**host lipid bodies**
^{13}C	feeding mode (F)	$F_{1,565}$ = 1031.004, p<0.0001*	$F_{1,140}$ = 108.896, p<0.0001*	$F_{1,140}$ = 1576.139, p<0.0001*	$F_{1,618}$ = 1257.353, p<0.0001*
	temperature acclimation (T)	$F_{1,565}$ = 100.112, p<0.0001*	$F_{1,140}$ = 164.544, p<0.0001*	$F_{1,140}$ = 286.319, p<0.0001*	$F_{1,618}$ = 72.467, p<0.0001*
	replicate (R)	$F_{2,564}$ = 7.305, p=0.0007*	$F_{2,139}$ = 4.487, p=0.0130*	$F_{2,139}$ = 82.625, p<0.0001*	$F_{2,617}$ = 2.838, p=0.0593
	F x T	$F_{1,563}$ = 0.457, p=0.4995	$F_{1,138}$ = 43.660, p<0.0001*	$F_{1,138}$ = 229.191, p<0.0001*	N/A
	T x R	$F_{2,561}$ = 11.542, p<0.0001*	$F_{2,136}$ = 8.538, p=0.0003*	$F_{2,136}$ = 3.231, p=0.0427*	$F_{2,614}$ = 13.504, p<0.0001*
	F x R	$F_{2,561}$ = 0.696, p=0.4993	$F_{2,136}$ = 7.723, p=0.0007*	$F_{2,136}$ = 58.039, p<0.0001*	$F_{2,614}$ = 13.814, p<0.0001*
	F x T x R	$F_{2,555}$ = 7.762, p=0.0005*	$F_{2,130}$ = 3.871, p=0.0233*	$F_{2,130}$ = 5.359, p=0.0058*	N/A
^{15}N	feeding mode (F)	$F_{1,565}$ = 495.822, p<0.0001*	$F_{1,140}$ = 649.590, p<0.0001*	$F_{1,140}$ = 2422.978, p<0.0001*	$F_{1,618}$ = 503.026, p<0.0001*
	temperature acclimation (T)	$F_{1,565}$ = 657.368, p<0.0001*	$F_{1,140}$ = 171.770, p<0.0001*	$F_{1,140}$ = 394.396, p<0.0001*	$F_{1,618}$ = 72.560, p<0.0001*
	replicate (R)	$F_{2,564}$ = 293.411, p<0.0001*	$F_{2,139}$ = 23.576, p<0.0001*	$F_{2,139}$ = 40.764, p<0.0001*	$F_{2,617}$ = 18.860, p<0.0001*
	F x T	$F_{1,520}$ = 507.797, p<0.0001*	$F_{1,138}$ = 166.140, p<0.0001*	$F_{1,138}$ = 388.653, p<0.0001*	$F_{1,616}$ = 43.136, p<0.0001*
	T x R	$F_{2,561}$ = 182.553, p<0.0001*	$F_{2,136}$ = 14.416, p<0.0001*	$F_{2,136}$ = 6.100, p=0.0029*	$F_{2,614}$ = 12.670, p<0.0001*
	F x R	$F_{2,561}$ = 41.064, p<0.0001*	$F_{2,136}$ = 22.471, p<0.0001*	$F_{2,136}$ = 40.183, p<0.0001*	$F_{2,614}$ = 21.014, p<0.0001*
	F x T x R	$F_{2,555}$ = 75.621, p<0.0001*	$F_{2,130}$ = 12.959, p<0.0001*	$F_{2,130}$ = 6.375, p=0.0023*	$F_{2,608}$ = 5.741, p=0.0034*

Table S4. Summary of NanoSIMS raw data

mode of nutrition	feeding acclimation	temperature	ROI	replicate	normalized ^{13}C APE [%]		normalized ^{15}N turnover [%]		ROI diameter ±SE [µm]	ROI area ±SE [µm^2]	N
					mean	SE	mean	SE			
autotrophic	unfed	ambient	Symbiont	A	5.3402	0.2158	2.2900	0.0738	7.8±0.3		52
autotrophic	unfed	ambient	Symbiont	G	5.9558	0.3149	2.0309	0.0582	6.7±0.2		50
autotrophic	unfed	ambient	Symbiont	I	5.0078	0.2399	1.5248	0.0659	7.0±0.2		62
autotrophic	unfed	ambient	Gastrodermis	A	1.5701	0.1957	0.1733	0.0171		354.7±30.1	11
autotrophic	unfed	ambient	Gastrodermis	G	1.6326	0.0908	0.2286	0.0176		280.4±18.7	10
autotrophic	unfed	ambient	Gastrodermis	I	0.9182	0.1378	0.0990	0.0160		349.2±28.7	13
autotrophic	unfed	ambient	Epidermis	A	0.0164	0.0056	0.0106	0.0015		586.2±42.4	11
autotrophic	unfed	ambient	Epidermis	G	0.0531	0.0033	0.0164	0.0005		391.6±29.1	10
autotrophic	unfed	ambient	Epidermis	I	0.0447	0.0058	0.0103	0.0012		508.6±57.1	13
autotrophic	unfed	ambient	Host lipid	A	8.0961	0.2871	0.2545	0.0239	2.7±0.2		61
autotrophic	unfed	ambient	Host lipid	G	7.8277	0.2201	0.2630	0.0243	2.6±0.2		55
autotrophic	unfed	ambient	Host lipid	I	8.1344	0.3755	0.2376	0.0301	2.7±0.2		43
autotrophic	unfed	ambient	ExLipid	A	9.9633	0.7970			1.5±0.1		6
autotrophic	unfed	ambient	ExLipid	G	10.6604	0.5773			1.4±0.2		6
autotrophic	unfed	ambient	ExLipid	I	8.9082	1.0636			1.8±0.3		9
autotrophic	unfed	high	Symbiont	A	5.3730	0.2738	1.9423	0.0505	8.1±0.3		47
autotrophic	unfed	high	Symbiont	G	3.9549	0.2129	1.3990	0.0441	7.4±0.3		59
autotrophic	unfed	high	Symbiont	I	4.2051	0.2158	1.9820	0.0655	7.0±0.3		46
autotrophic	unfed	high	Gastrodermis	A	1.5517	0.1656	0.1488	0.0085		282.2±17.5	14
autotrophic	unfed	high	Gastrodermis	G	0.5411	0.0558	0.0514	0.0061		301.3±14.6	22
autotrophic	unfed	high	Gastrodermis	I	0.6657	0.1052	0.0844	0.0103		284.2±18.9	16
autotrophic	unfed	high	Epidermis	A	0.0326	0.0026	0.0060	0.0008		651.5±37.6	14
autotrophic	unfed	high	Epidermis	G	0.0532	0.0031	0.0079	0.0004		595.1±17.5	22
autotrophic	unfed	high	Epidermis	I	0.0242	0.0051	0.0089	0.0009		557.9±26.6	16

autotrophic	unfed	high	Host lipid	A	9.1415	0.3552	0.2410	0.0246	3.0±0.2		46
autotrophic	unfed	high	Host lipid	G	7.3055	0.3403	0.1502	0.0153	2.2±0.1		38
autotrophic	unfed	high	Host lipid	I	5.5690	0.2101	0.2093	0.0283	2.4±0.2		35
autotrophic	unfed	high	ExLipid	A	10.0562	0.5380			1.7±0.1		11
autotrophic	unfed	high	ExLipid	G	9.7270	0.4242			1.6±0.1		23
autotrophic	unfed	high	ExLipid	I	8.3224	0.5312			2.0±0.4		17
autotrophic	fed	ambient	Symbiont	A	5.9965	0.3120	2.9410	0.0756	7.7±0.3		46
autotrophic	fed	ambient	Symbiont	G	6.5324	0.3399	1.7447	0.0661	7.2±0.3		38
autotrophic	fed	ambient	Symbiont	I	5.7702	0.2898	1.3445	0.0685	6.7±0.2		56
autotrophic	fed	ambient	Gastrodermis	A	1.0711	0.1616	0.1788	0.0169		342.7±23.0	14
autotrophic	fed	ambient	Gastrodermis	G	2.1113	0.2341	0.1317	0.0199		309.7±23.1	8
autotrophic	fed	ambient	Gastrodermis	I	1.5983	0.3037	0.1203	0.0192		319.3±30.3	8
autotrophic	fed	ambient	Epidermis	A	0.0205	0.0025	0.0105	0.0010		606.2±29.5	14
autotrophic	fed	ambient	Epidermis	G	0.0594	0.0048	0.0105	0.0006		437.9±16.0	8
autotrophic	fed	ambient	Epidermis	I	0.0965	0.0098	0.0154	0.0011		403.9±25.6	8
autotrophic	fed	ambient	Host lipid	A	8.3314	0.4160	0.3502	0.0326	2.9±0.2		43
autotrophic	fed	ambient	Host lipid	G	8.4992	0.4041	0.1839	0.0288	3.2±0.2		39
autotrophic	fed	ambient	Host lipid	I	7.1330	0.3452	0.1861	0.0201	2.8±0.2		46
autotrophic	fed	ambient	ExLipid	A	9.6460	0.8327			1.4±0.3		4
autotrophic	fed	ambient	ExLipid	G	11.3072	0.5382			1.4±0.1		8
autotrophic	fed	ambient	ExLipid	I	9.2273	0.3115			1.7±0.2		11
autotrophic	fed	high	Symbiont	A	5.2896	0.2519	2.2841	0.0600	8.6±0.3		45
autotrophic	fed	high	Symbiont	G	4.7228	0.2627	1.4849	0.0391	7.7±0.3		51
autotrophic	fed	high	Symbiont	I	4.3107	0.2421	1.6854	0.0923	8.0±0.3		57
autotrophic	fed	high	Gastrodermis	A	0.5127	0.0788	0.0879	0.0097		334.9±22.2	18
autotrophic	fed	high	Gastrodermis	G	0.5411	0.0719	0.0773	0.0061		297.1±17.8	16
autotrophic	fed	high	Gastrodermis	I	1.2691	0.0917	0.1533	0.0278		440.8±36.7	10
autotrophic	fed	high	Epidermis	A	0.0208	0.0044	0.0081	0.0005		586.9±36.3	18
autotrophic	fed	high	Epidermis	G	0.0378	0.0035	0.0086	0.0009		610.5±38.1	16
autotrophic	fed	high	Epidermis	I	0.0308	0.0045	0.0082	0.0020		563.5±54.4	10

autotrophic	fed	high	Host lipid	A	7.6561	0.3385	0.3161	0.0445	2.5±0.2		33
autotrophic	fed	high	Host lipid	G	5.9577	0.4374	0.1491	0.0228	2.3±0.2		33
autotrophic	fed	high	Host lipid	I	6.3118	0.3526	0.2374	0.0360	2.9±0.2		52
autotrophic	fed	high	ExLipid	A	9.6369	1.1707			1.6±0.2		8
autotrophic	fed	high	ExLipid	G	7.7264	0.5387			1.6±0.1		17
autotrophic	fed	high	ExLipid	I	10.0416	1.0836			1.8±0.2		11
heterotrophic	fed	ambient	Symbiont	A	2.0436	0.1055	7.2170	0.2655	7.5±0.2		43
heterotrophic	fed	ambient	Symbiont	G	2.8629	0.1190	4.0831	0.1004	6.9±0.3		44
heterotrophic	fed	ambient	Symbiont	I	1.1887	0.0614	3.0410	0.1172	6.4±0.2		51
heterotrophic	fed	ambient	Gastrodermis	A	4.3194	0.3963	8.8649	0.5866		416.3±20.9	16
heterotrophic	fed	ambient	Gastrodermis	G	3.5699	0.2611	5.0863	0.4006		365.9±22.8	11
heterotrophic	fed	ambient	Gastrodermis	I	3.6260	0.3346	6.3800	0.4158		432.5±30.5	7
heterotrophic	fed	ambient	Epidermis	A	0.7482	0.0329	1.8820	0.0873		695.0±39.4	16
heterotrophic	fed	ambient	Epidermis	G	0.9370	0.0478	1.5785	0.0658		581.5±16.1	11
heterotrophic	fed	ambient	Epidermis	I	1.1653	0.0554	2.0240	0.0665		411.5±52.8	7
heterotrophic	fed	ambient	Host lipid	A	1.5542	0.1669	1.6051	0.1278	3.0±0.2		77
heterotrophic	fed	ambient	Host lipid	G	3.3400	0.2487	2.1631	0.4084	2.4±0.1		76
heterotrophic	fed	ambient	Host lipid	I	1.9106	0.1726	2.4107	0.4027	2.6±0.2		70
heterotrophic	fed	ambient	Gastrohotspot	A	31.2981	1.2941	40.7237	0.9787	1.25±0.03		249
heterotrophic	fed	ambient	Gastrohotspot	G	20.3818	1.3333	24.9078	1.0269	1.17±0.03		125
heterotrophic	fed	ambient	Gastrohotspot	I	26.4932	1.8968	34.9988	1.3792	1.16±0.03		116
heterotrophic	fed	ambient	Epihotspot	A	6.2617	0.5030	18.1173	0.7986	1.11±0.04		56
heterotrophic	fed	ambient	Epihotspot	G	8.0850	0.5327	13.5130	0.5113	0.79±0.02		76
heterotrophic	fed	ambient	Epihotspot	I	7.3076	0.8690	10.4921	0.9813	0.86±0.03		45
heterotrophic	fed	high	Symbiont	A	0.8874	0.0654	2.2501	0.0900	8.4±0.3		44
heterotrophic	fed	high	Symbiont	G	0.5037	0.0424	0.5704	0.0267	7.1±0.2		40
heterotrophic	fed	high	Symbiont	I	1.2311	0.0735	2.5843	0.1024	7.0±0.3		52
heterotrophic	fed	high	Gastrodermis	A	1.0754	0.0788	2.1358	0.1795		353.0±20.1	16
heterotrophic	fed	high	Gastrodermis	G	0.5676	0.0673	0.8086	0.0638		264.7±23.8	10
heterotrophic	fed	high	Gastrodermis	I	2.1924	0.2540	3.9066	0.3936		337.8±22.1	8

heterotrophic	fed	high	Epidermis	A	0.2382	0.0222	0.6607	0.0329		591.7±37.0	16
heterotrophic	fed	high	Epidermis	G	0.2916	0.0444	0.4440	0.0195		456.0±22.0	10
heterotrophic	fed	high	Epidermis	I	0.7573	0.0478	1.2524	0.0312		531.5±34.1	8
heterotrophic	fed	high	Host lipid	A	0.6203	0.1218	0.4661	0.0415	2.7±0.1		72
heterotrophic	fed	high	Host lipid	G	0.6741	0.1669	0.2834	0.0223	2.3±0.1		29
heterotrophic	fed	high	Host lipid	I	1.4171	0.1564	1.1818	0.0919	2.4±0.1		50
heterotrophic	fed	high	Gastrohotspot	A	14.1724	1.7751	17.1550	1.6742	1.12±0.03		80
heterotrophic	fed	high	Gastrohotspot	G	4.6700	1.7513	5.5404	1.2176	1.14±0.08		25
heterotrophic	fed	high	Gastrohotspot	I	14.7748	1.5678	21.6888	1.2709	1.09±0.04		68
heterotrophic	fed	high	Epihotspot	A	1.3035	0.3559	5.2883	0.2436	1.06±0.05		44
heterotrophic	fed	high	Epihotspot	G	1.5046	0.2881	3.9654	0.2506	0.96±0.03		63
heterotrophic	fed	high	Epihotspot	I	4.4311	0.4317	8.0752	0.5120	0.95±0.04		35

Table S5. Statistical results of the Two-Way ANOVA on the effects of temperature and replicate on the heterotrophic fixation of carbon and nitrogen from a brine shrimp for each region of interest (cf. Fig. 6: Het [F]). Asterisks indicate statistical significance.

element	compartment	Symbiont	Gastrodermis	Epidermis	Host lipid bodies
^{13}C	replicate (R)	$F_{2,271}$ = 17.158, p<0.0001*	$F_{2,65}$ = 4.183, p=0.0198*	$F_{2,65}$ = 63.356, p<0.0001*	$F_{2,371}$ = 11.267, p<0.0001*
	temperature acclimation (T)	$F_{1,272}$ = 298.391, p<0.0001*	$F_{1,66}$ = 113.063, p<0.0001*	$F_{1,66}$ = 244.079, p<0.0001*	$F_{1,372}$ = 69.298 p<0.0001*
	R x T	$F_{2,268}$ = 109.604, p<0.0001*	$F_{2,62}$ = 4.895, p=0.0106*	$F_{2,62}$ = 3.745, p=0.0291*	$F_{2,368}$ = 14.018, p<0.0001*
^{15}N	replicate (R)	$F_{2,271}$ = 167.730, p<0.0001*	$F_{2,65}$ = 21.822, p<0.0001*	$F_{2,65}$ = 37.411, p<0.0001*	$F_{2,371}$ = 10.174, p<0.0001*
	temperature acclimation (T)	$F_{1,272}$ = 715.600, p<0.0001*	$F_{1,66}$ = 155.285, p<0.0001*	$F_{1,66}$ = 365.760, p<0.0001*	$F_{1,372}$ = 84.389 p<0.0001*
	R x T	$F_{2,268}$ = 150.906, p<0.0001*	$F_{2,62}$ = 12.587, p<0.0001*	$F_{2,62}$ = 5.742, p=0.0052*	$F_{2,368}$ = 5.387, p=0.0049*
